# STAT2 Is a Pervasive Cytokine Regulator due to Its Inhibition of STAT1 in Multiple Signaling Pathways

**DOI:** 10.1371/journal.pbio.2000117

**Published:** 2016-10-25

**Authors:** Johnathan Ho, Christin Pelzel, Andreas Begitt, Maureen Mee, Hany M. Elsheikha, David J. Scott, Uwe Vinkemeier

**Affiliations:** 1 School of Life Sciences, Queen’s Medical Centre, University of Nottingham, Nottingham, United Kingdom; 2 School of Veterinary Medicine and Science, University of Nottingham, Loughborough, United Kingdom; 3 ISIS Spallation Neutron and Muon Source, Rutherford Appleton Laboratory, Didcot, United Kingdom; 4 Research Complex at Harwell, Rutherford Appleton Laboratory, Didcot, United Kingdom; 5 School of Biosciences, University of Nottingham, Nottingham, United Kingdom; Whitehead Institute for Biomedical Research, United States of America

## Abstract

STAT2 is the quintessential transcription factor for type 1 interferons (IFNs), where it functions as a heterodimer with STAT1. However, the human and murine STAT2-deficient phenotypes suggest important additional and currently unidentified type 1 IFN-independent activities. Here, we show that STAT2 constitutively bound to STAT1, but not STAT3, via a conserved interface. While this interaction was irrelevant for type 1 interferon signaling and STAT1 activation, it precluded the nuclear translocation specifically of STAT1 in response to IFN-γ, interleukin-6 (IL-6), and IL-27. This is explained by the dimerization between activated STAT1 and unphosphorylated STAT2, whereby the semiphosphorylated dimers adopted a conformation incapable of importin-α binding. This, in turn, substantially attenuated cardinal IFN-γ responses, including MHC expression, senescence, and antiparasitic immunity, and shifted the transcriptional output of IL-27 from STAT1 to STAT3. Our results uncover STAT2 as a pervasive cytokine regulator due to its inhibition of STAT1 in multiple signaling pathways and provide an understanding of the type 1 interferon-independent activities of this protein.

## Author Summary

For more than a quarter century, we have known that STAT1 and STAT2 are essential for the classic host immune defense system against viral infections known as the type 1 interferon response. While STAT1 has since been assigned multiple additional roles, STAT2 is thought to function exclusively as the principal partner of STAT1 in the type 1 interferon system. However, patients and animals that are deficient in STAT2 show a surprisingly varied and sometimes subtle phenotype not fully accounted for by the known functions of this protein. Our investigations reveal an entirely novel facet of STAT2 action, namely as an innate inhibitor of STAT1 in its multiple biological roles. We identify the molecular mechanism of STAT1 inhibition and generate a novel biological tool with which we can dissociate STAT2’s activating and inhibitory effects on STAT1. We use this tool to show that STAT2 has major roles beyond antiviral protection, for example, in regulating cell proliferation and immune cell functions, as well as in killing intracellular parasites. These findings considerably expand our knowledge of STAT2 biology and necessitate a reassessment of regulatory mechanisms central to innate immunity and the therapeutic use of interferons.

## Introduction

Cytokines are a structurally and functionally diverse group of small proteins that function as extracellular signaling molecules. They control all aspects of immune cell biology including development, differentiation, activation, and death and orchestrate the functioning of the entire immune system. Studies of patients and animals that lack cytokines or their receptors have assigned distinct activities to individual cytokines, while simultaneously revealing a more complex scenario with extensive functional overlap [1]. The molecular understanding of how the balance is struck between specificity and redundancy is incomplete and a central problem in cytokine biology. This is because there are over 50 different cytokines but only seven signal transducer and activator of transcription (STAT) proteins that deliver the signals, namely STAT1-4, 5A, 5B, and 6 [2]. A cytokine binding to its receptor triggers a phosphorylation cascade resulting in the modification of STAT proteins at a single tyrosine residue, an event also called STAT activation [3]. STAT activation is associated with the rearrangement of preformed dimers from an antiparallel conformation to a parallel conformation, whereby interactions between the aminoterminal domains (N domains) are critically important [4,5]. Transition to the parallel conformation has important consequences; transforming the STATs into sequence-specific transcription factors that can bind DNA with high affinity [6]. Moreover, and contrary to the antiparallel dimers, which are carrier-independent nucleocytoplasmic shuttling proteins, activated dimers require transport factors termed importins for nuclear translocation and accumulation [7]. STAT1 is the major STAT protein activated in response to IFN-γ, the sole type 2 IFN [3]. STAT1 mediates many of the IFN-γ functions through the direct activation of genes such as those that are essential for host defense against intracellular bacteria and parasites [8]. Given the self-damaging potential of these activities, IFN-γ signaling requires tight control, such as feedback inhibition of Janus kinases (JAKs) through the up-regulation of suppressor of cytokine signalling (SOCS) proteins [9]. STAT1 is activated by multiple cytokines in addition to IFN-γ, including interleukin-27 and -6 (IL-27 and IL-6), and often in conjunction with the closely related STAT3 [10]. STAT1 and STAT3 have very similar DNA sequence preferences, yet their transcriptomes are distinct and overlap only partially [11,12]. In fact, STAT1 and STAT3 oppose each other in many biological processes, but little is known about mechanisms that determine their relative contributions to the overall transcription output.

In contrast to STAT1 and STAT3, which take part in multiple signaling pathways, STAT2 is considered to be involved in only a single intracellular pathway, which emanates from the receptors of type 1 and type 3 IFNs [13]. In further contrast to the other STAT proteins, which primarily assemble homodimers upon cytokine stimulation, STAT2 exclusively forms heterodimers with concurrently activated STAT1, which associate with another DNA-binding protein, IRF9, and form the transcription factor interferon-stimulated gene factor 3 (ISGF3) [3]. STAT2 deficiency in both patients and animals accordingly precludes the up-regulation of hundreds of genes in response to IFN-α or IFN-β and the other type 1/3 IFNs, many of which have direct and indirect antiviral activity, and results in a marked susceptibility to viral infections [14,15]. Other aspects of STAT2 deficiency are unexplained, though, such as the exacerbation of experimental sepsis observed with STAT2-deficient mice, given that genetic deletion of other components of type 1 IFN signaling results in the opposite phenotype, namely protection against sepsis [16,17]. These, and other, data suggest additional roles for STAT2 in cytokine signaling beyond ISGF3 and type 1/3 IFNs. In this context, we now report that STAT2 is a critical regulator for multiple STAT1-activating cytokines. We provide detailed insight into the molecular underpinnings, and examine the consequences for key cytokine activities both in vitro and in vivo. Our experiments showed that STAT2 attenuated crucial immunomodulatory and antimicrobial effector functions of IFN-γ; and in IL-27-mediated transcription, STAT2 shifted the balance away from STAT1 to STAT3. Thus, in addition to its well-known role as an IFN-activated transcription factor, STAT2 has pervasive activation-independent activities as a STAT1 negative regulator in multiple signaling pathways.

## Results

### STAT2 Binds to STAT1 through Exceptionally Strong N Domain Interactions

**Fig 1:**
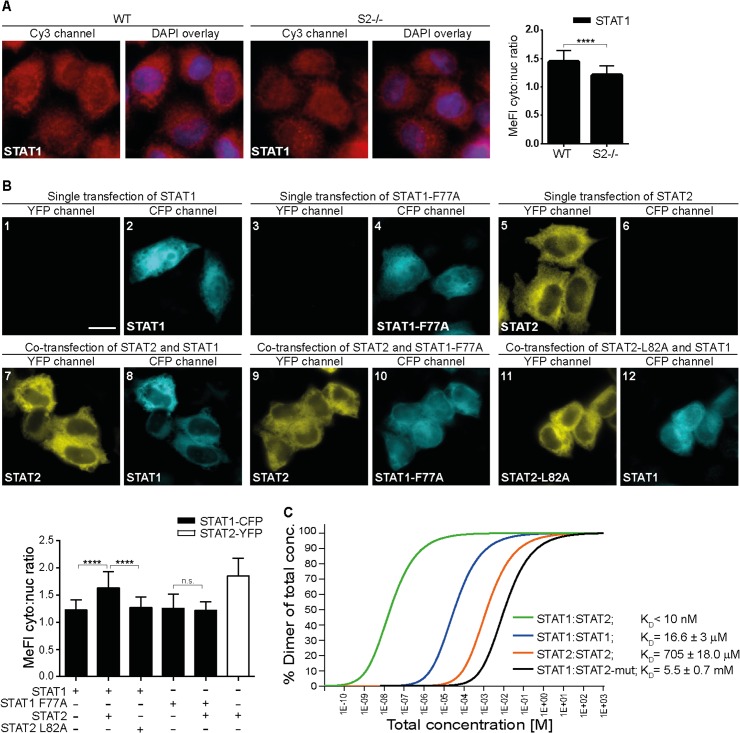
STAT2 binds STAT1 via high affinity N Domain interactions. **(A)** Stat2-deficient human U6A cells (S2-/-) and parental 2fTGH cells (WT) were fixed and processed for immunostaining using STAT1 antibody, nuclear counterstaining was with DAPI. Shown are deconvolution fluorescence micrographs. The corresponding bar graphs show STAT1 cytoplasmic/nuclear signal ratios and standard deviation (s.d.) produced using line scanning to acquire mean fluorescence intensities (MeFIs) in cytoplasm and nucleus. Scale bar = 15 μm. **(B)** STAT1-CFP and STAT2-YFP variants were singly or coexpressed in HeLa cells and their localization was determined by deconvolution fluorescence microscopy. Bar graphs show cytoplasmic/nuclear signal ratios for STAT1 (black bars) and STAT2 (white bar) acquired as described in (A). Scale bar = 15 μm. **(C)** Equilibration sedimentation analyses of homo- and heterotypic associations of STAT1 and STAT2 recombinant N domains. Equilibrium dissociation constant (K_D_); (STAT2-mut) STAT2 LL81,82AA. See S1 Data for raw data.

During studies of STAT protein self-association, we noticed a more prominent cytoplasmic steady-state localization of STAT1 in cells that expressed STAT2 compared to cells that lacked it (Fig 1A). This observation was recapitulated in cells overexpressing STAT1 and STAT2 tagged with cyan fluorescent protein (CFP) and yellow fluorescent protein (YFP), respectively. The tagged STAT1 distributed throughout the cell (Fig 1B, panels 1–4), while separately expressed STAT2 accumulated in the cytoplasm (Fig 1B, panels 5 and 6). Coexpression of STAT1 and STAT2 demonstrated the cytoplasmic redistribution of STAT1 (Fig 1B, panels 7 and 8). A similar redistribution upon STAT2 overexpression was not observed with the two STAT proteins most closely related to STAT1, STAT3 and STAT4 (S1 Fig). The results suggested binding interactions specifically between STAT1 and STAT2. Both are nucleocytoplasmic shuttling proteins, whereby STAT2 accumulates in the cytoplasm due to efficient nuclear export signal (NES)-mediated transport by the transportin CRM1 [18,19]. In line with this reasoning, inactivation of the STAT2 NES, either by genetic removal of the NES-containing C terminal transactivation domain or by chemical inhibition of the NES receptor protein CRM1 [20], resulted in similar pancellular distribution of both STAT2 and STAT1 (S2 Fig). We then asked whether mutational inactivation of the interface that mediates the homodimerization of unphosphorylated (aka latent) STAT1, namely replacement with alanine of phenylalanine 77 in the N domain [21], affected its colocalization with STAT2. This was the case, as the mutant STAT1 failed to colocalize with STAT2 (Fig 1B, panels 9 and 10). We then did the reverse experiment, i.e., we coexpressed wild type (WT) STAT1 and mutant STAT2 that harbored alanine in the STAT1 F77 homologous position leucine 82 (Fig 1B, panels 11 and 12) or the functionally equivalent double mutation LL81,82AA (S3 Fig). Strikingly, the two STAT2 mutants phenocopied STAT1-F77A. The mutated STAT2 retained cytoplasmic accumulation, but STAT1 failed to redistribute. We concluded that STAT1 homodimers and its heterodimers with STAT2 assembled via identical N domain-mediated interactions.

Quantitative data on the heterodimerization of STAT proteins or their N domains were not previously available. Data for homodimers demonstrate that STAT N domains differ considerably in their interaction strengths, and that this measure can be used as a proxy for dimerization of the full-length STAT proteins [5,22]. We therefore purified N domains of STAT1 and STAT2 and determined dissociation constants for homo- and heterodimers using analytical ultracentrifugation (Fig 1C). STAT2 homodimers were found to associate at high micromolar concentrations, indicating weak protein interactions (Fig 1C). For comparison, homodimers of STAT1 N domain were about 50-fold stronger (Fig 1C), in agreement with previous measurements [22]. A very different picture emerged when STAT2 heterodimerization was assessed. Heterodimerization between STAT2 and STAT1 was exceptional, namely at least 1,000 times stronger than STAT1 homodimers. In sharp contrast, the LL81,82AA-mutated STAT2 N domain that abrogated colocalization with STAT1 in living cells was essentially devoid of dimerization activity at the concentrations tested (Fig 1C). These experiments confirmed that identical N domain interactions stabilized the homodimers and heterodimers of STAT1, whereby the heterotypic interactions were stronger by at least three orders of magnitude.

### Latent STAT2 Inhibits the IFN-γ-Induced Nuclear Import and DNA Binding of STAT1

**Fig 2:**
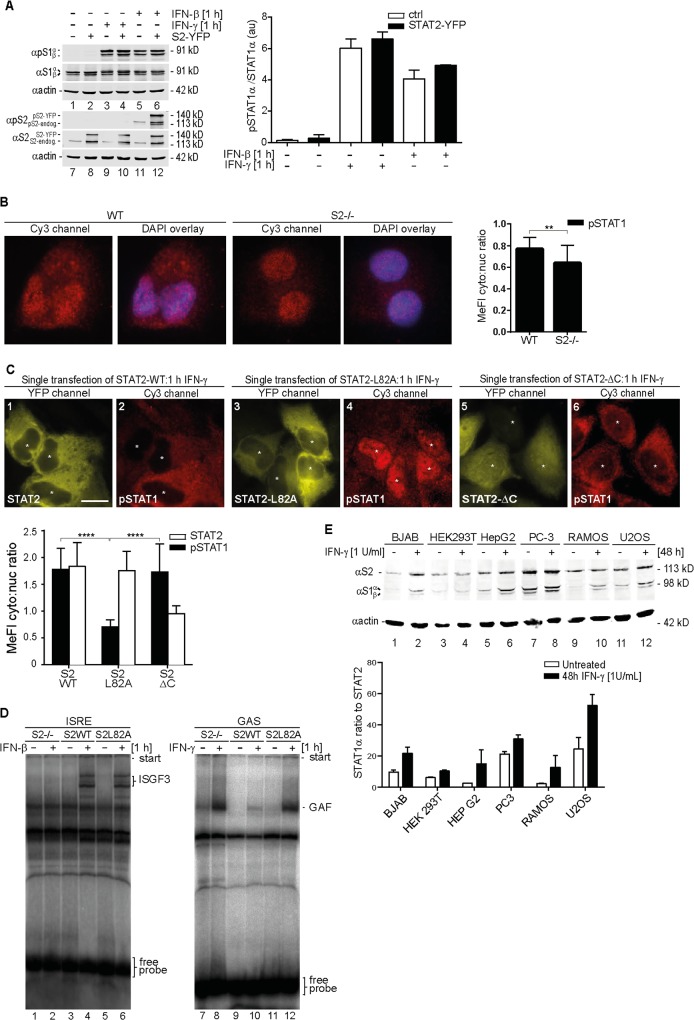
STAT2 inhibits nuclear translocation and DNA-binding of IFN-γ-activated STAT1. **(A)** Quantitative western blotting with extracts from HeLa cells expressing STAT2-YFP or empty vector (ctrl). The positions of latent (α) and activated (αp) STAT1 (S1) and STAT2 (S2) are indicated. Transfected (-YFP) and endogenous (-endog.) STAT2 are discriminated. kD, kilo Dalton. Bar graph shows mean and s.d. **(B)** STAT2-deficient human U6A cells (S2-/-) and parental 2fTGH cells (WT) were treated for 1 h with 1 U/ml IFN-γ, fixed and processed for immunostaining using anti-phospho-Y701 STAT1 antibody, nuclear counterstaining was with DAPI. Shown are deconvolution fluorescence micrographs. The corresponding bar graphs show activated STAT1 cytoplasmic/nuclear signal ratios and s.d. produced using line scanning to acquire MeFIs in cytoplasm and the nucleus. Scale bar = 15 μm. **(C)** as (B), with HeLa cells overexpressing WT STAT2-YFP or variants L82A and truncated STAT2 (ΔC) treated for 1 h with 50 U/ml IFN-γ. (*) denotes cell nuclei. Bar graphs show cytoplasmic/nuclear signal ratios and s.d. for phospho-Y701 STAT1 in the presence of the indicated STAT2 variants. Scale bar = 15 μm. **(D)** Electrophoretic mobility shift assays (EMSAs) with radiolabeled interferon-stimulated response element (ISRE) (left) and gamma interferon-activated site (GAS) (right) probes to detect ISGF3 and gamma interferon-activated factor (GAF), respectively, the positions of which and of the free probes are indicated. Extracts from parental (S2-/-) and stably STAT2-reconstituted (S2 WT or S2 L82A) human U6A cells were used. **(E)** Quantitative western blotting results with extracts from the indicated cell lines showing the positions of STAT1 splice variants (αS1α/β) and STAT2 (αS2). Bar graph shows mean and s.d. See S1 Data for raw data.

Next, we evaluated the effects of STAT2 on the IFN-dependent functions of STAT1. Western blotting experiments using cell extracts from control and STAT2-overexpressing HeLa cells confirmed that IFN-γ induced the tyrosine phosphorylation of STAT1, but not STAT2, (Fig 2A, lanes 3, 4, 9, and 10), whereas IFN-β activated both STATs (Fig 2A, lanes 5, 6, 11, and 12). STAT2 overexpression, however, was without negative consequences for STAT1 activation by both IFN-γ (Fig 2A, lanes 3 and 4) and IFN-β (lanes 5 and 6). Yet, despite undiminished activation, the IFN-γ-induced nuclear translocation of STAT1 was significantly diminished in the presence of STAT2 at native protein levels (Fig 2B). To explore the underlying molecular mechanisms, we transiently expressed STAT2 variant proteins in HeLa cells and observed the nuclear translocation of endogenous STAT1 in response to IFN-γ. Recombinant STAT2, like the endogenous STAT2, inhibited STAT1 nuclear accumulation (Fig 2C, panels 1 and 2), in agreement with a previous observation by Julkunen and colleagues [23]. The same result was obtained with C-terminally truncated STAT2 (aa 1–702) lacking NES activity, demonstrating that cytoplasmic retention of activated STAT1 is independent of STAT2 nuclear export (Fig 2C, panels 5 and 6). In stark contrast, the STAT2-L82A mutant that cannot bind to STAT1 failed to prevent the IFN-γ-induced nuclear translocation of activated STAT1 (Fig 2C, panels 3 and 4), thus recapitulating its effect on the latent STAT1 (Fig 1B, panels 11 and 12). STAT1 nuclear import in response to type 1 IFN, in contrast, was unaffected by disrupted STAT1:STAT2 heterodimerization (S4A Fig).

Another major consequence of STAT1 activation is its ability to bind specific DNA sequences with high affinity, namely to the interferon-stimulated response element (ISRE) as part of ISGF3, and to the gamma interferon-activated site (GAS) as a homodimer (gamma interferon-activated factor; GAF) [3]. To test whether STAT2 interferes with STAT1 DNA binding, we used electrophoretic mobility shift assays (EMSAs) with extracts from STAT2-deficient human U6A fibrosarcoma cells and stable derivatives expressing WT STAT2 or mutant STAT2-L82A (Fig 2D, S5 Fig). Of note, whole cell extracts normalized for activated STAT1 were used to account for the nuclear import inhibition caused by STAT2. As shown in Fig 2D, STAT2 was required for IFN-β-induced ISGF3 formation, whereby WT STAT2 and the STAT1-binding mutant were indistinguishable (lanes 4 and 6). In contrast, the IFN-γ-induced STAT1 homodimer (GAF) activity expectedly did not require STAT2 (lane 8), yet a substantial difference was observed between the WT and mutant STAT2-L82A-containing extracts. WT STAT2 resulted in ≈75% reduced GAF activity (lanes 8 and 10), whereas the mutant STAT2 left GAF essentially unchanged (lanes 8 and 12).

Since increasing the concentration of STAT2 strongly affected STAT1 activities, we determined their abundance (Fig 2E, S6 Fig). In all cell lines tested, the full-length STAT1α was present in molar excess over STAT2, albeit to a considerably varying degree (≈2- to 25-fold). As both STATs are IFN-stimulated genes (ISGs), prolonged treatment with a low IFN-γ concentration (priming) expectedly increased their concentration (Fig 2E) [24], whereby STAT1α’s excess over STAT2 was further heightened 2- to 5-fold. We concluded that a sizable and dynamic STAT1 fraction was bound to unphosphorylated STAT2 before IFN treatment. Since STAT2 remained bound to STAT1 in response to IFN-γ, but was not phosphorylated, semiphosphorylated heterodimers resulted that precluded the subsequent nuclear import and DNA binding of the activated STAT1.

### STAT2 Inhibits the Nuclear Import Specifically of STAT1 in Multiple Signaling Pathways

**Fig 3:**
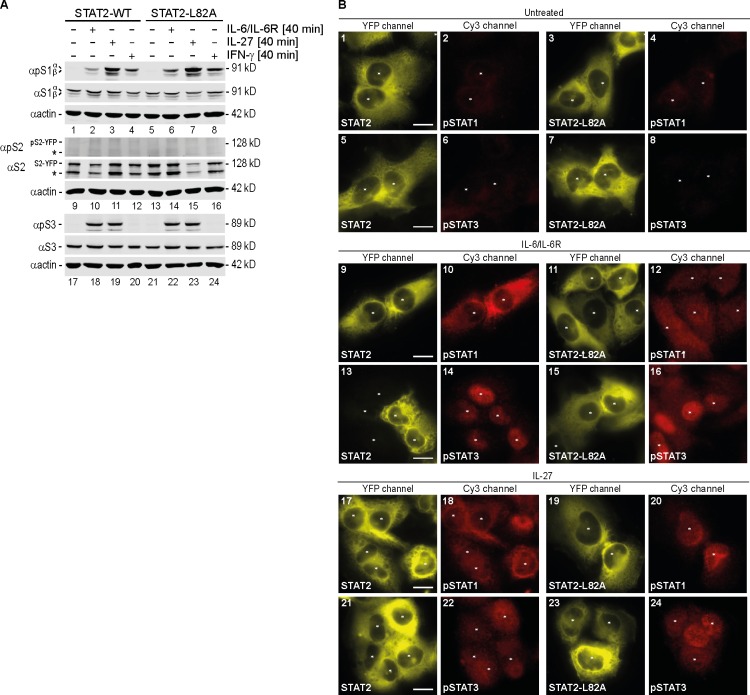
STAT2 inhibits specifically STAT1 in response to both IL-27 and IL-6. **(A)** Western blot indicating the positions of latent (α) and activated (αp) STAT1α/β (S1α/β), STAT2 (S2), and STAT3 (S3). STAT2-YFP (S2-YFP) and its YFP-free degradation product (*) are discriminated. Treatment of cells was without or with IFN-γ, IL-27, or combined IL-6 and soluble IL-6 receptor (IL-6/IL-6R). **(B)** Deconvolution microscopy detecting activated STAT1 (anti-phospho-Y701 STAT1 antibody) and STAT3 (anti-phospho-Y705 STAT3 antibody) in STAT2-YFP-reconstituted U6A cells left untreated (top) or cotreated with IL-6/soluble IL-6 receptor (middle) or with IL-27 (bottom). (*) denotes cell nuclei. Scale bar = 15 μm.

To examine if STAT2’s nuclear import inhibition was specific for STAT1 or IFN-γ signaling, we treated WT STAT2- and STAT2-L82A-reconstituted U6A cells with IL-6 or IL-27, two cytokines that signal predominantly via STAT1 and STAT3. As shown in Fig 3A, IL-6 and IL-27 activated both STAT1 (lanes 1–3 and 5–7) and STAT3 (lanes 17–19 and 21–23), whereas IFN-γ activated predominantly STAT1 (lanes 4, 8, 20, 24). Moreover, the tyrosine phosphorylation of both STAT1 and STAT3 was indistinguishable in cells expressing WT STAT2 or its STAT1-binding mutant. STAT2, on the other hand, remained unphosphorylated in response to all three cytokines (lanes 9–16). We then observed the intracellular distribution of STAT1, STAT2, and STAT3 with fluorescence microscopy before and after the treatment with IL-6 or IL-27. In line with their lack of activation (Fig 3A), the two STAT2 variants showed unaltered cytoplasmic accumulation under all conditions (Fig 3B; YFP-channel). Activated STAT1 and STAT3 were detectable with their respective anti-phosphorylated tyrosine antibody in response to IL-6 (middle panels) and IL-27 (bottom panels), which too agreed with the western blotting results (Fig 3A). The activated STAT3 entered the nucleus in the presence of WT as well as mutated STAT2 (Fig 3B, panels 14, 16, 22, and 24). The IL-27- and IL-6-activated STAT1, in contrast, entered the nucleus in the presence of mutated STAT2 (panels 12, 20), but not WT STAT2 (panels 10, 18), similar to the results with IFN-γ (Fig 2B). Thus, STAT2 nuclear import inhibition was specific for STAT1 but not linked to a specific cytokine stimulus.

### Semiphosphorylated STAT1:STAT2 Heterodimers Adopt Antiparallel Conformation Incompatible with Importin-α5 Binding

**Fig 4:**
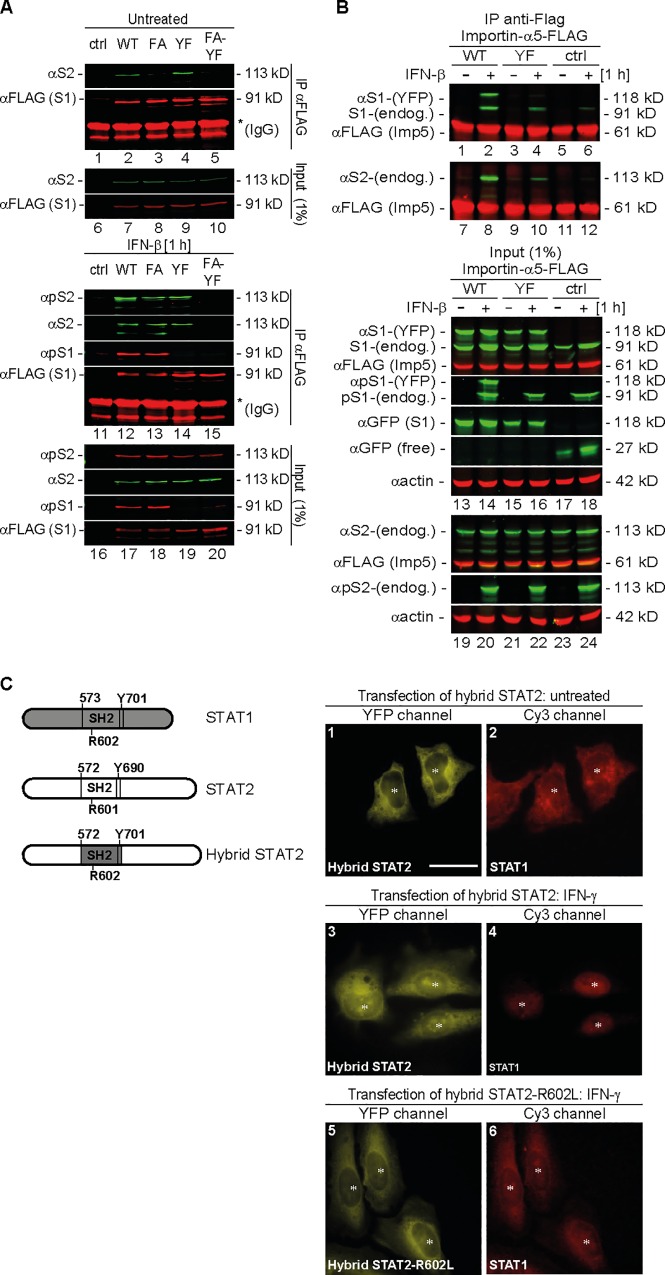
Dimers between latent STAT2 and activated STAT1 adopt an antiparallel conformation incompatible with importin-mediated nuclear import. **(A, B)** Immunoblots of precipitates (IP) and cell extracts (input) from immunoprecipitation experiments using anti-FLAG beads and HEK 293T cell extracts. Multicolor imaging results are shown, obtained by the concurrent application of up to three antibodies. **(A)** Cells were cotransfected with STAT2 and FLAG-tagged STAT1 variants (WT; F77A, FA; Y701F, YF; double mutant, FA-YF) or empty vector (ctrl) and left untreated (top) or treated with IFN-β (bottom). The positions of FLAG-tag (αFLAG) and latent (α) and activated (αp) STAT1 (S1) and STAT2 (S2) are indicated. **(B)** Cells were cotransfected with FLAG-tagged importin-α5 and YFP-tagged STAT1 (WT) or Y701F (YF) mutant or empty vector (ctrl) and treated without or with IFN-β. Labelling performed as in (A). Transfected (-YFP) and endogenous (-endog.) STATs are discriminated. Native YFP (free) and its STAT1 fusion (S1) were detected with anti-GFP antibody. (*) cross-reacting IgG. Note coprecipitation of the endogenous STAT1 in lanes 2, 4, and 6, i.e., with the IFN-β-treated extracts. **(C)** Schematic displaying STAT1, STAT2, and hybrid STAT2 structure accompanying widefield microscopy detecting the endogenous STAT1 (anti-STAT1 C terminus antibody decorated with Cy3) in cells expressing YFP-tagged hybrid STAT2. (*) denotes cell nucleus. Scale bar = 15 μm.

Activated STAT1 homodimers require the parallel conformation for nuclear import and DNA binding [6,21]. The respective requirements of the heterodimers were unknown. To understand STAT2 functioning as an import inhibitor of activated STAT1, we performed STAT2 coimmunoprecipitation assays with FLAG-tagged WT STAT1 and three STAT1 mutants deficient either in tyrosine phosphorylation (Y701F), or specifically antiparallel dimerization (F77A), or both (F77A, Y701F), to infer the conformation of unphosphorylated, semiphosphorylated, and fully phosphorylated STAT1:STAT2 heterodimers in vivo (Fig 4A). In cell extracts from IFN-untreated cells (Fig 4A, lanes 1–10), STAT2 coprecipitated with WT STAT1 and STAT1-Y701F (lanes 2, 4), but not STAT1-F77A and the STAT1-F77A, Y701F double mutant (lanes 3, 5). Thus, unphosphorylated STAT1 heterodimers, like the homodimers, required N domain interactions. To examine heterodimerization of the phosphorylated STATs, we repeated the experiment with extracts from IFN-ẞ-treated cells (Fig 4A, lanes 11–20). In this experiment, IFN-ẞ was used rather than IFN-γ in order to generate fully tyrosine-phosphorylated heterodimers as the positive control. As expected, the tyrosine-phosphorylated STAT2 coprecipitated with tyrosine-phosphorylated WT STAT1 (lane 12). Moreover, but contrary to the unphosphorylated situation (lane 3), tyrosine-phosphorylated STAT2 coprecipitated similarly well with the activated STAT1-F77A dimerization mutant (lane 13), presumably through mutual src kinase homology 2 (SH2):phosphotyrosine interactions. Heterodimerization also occurred if one partner was unphosphorylated (STAT1-Y701F) (lane 14), but not if the double mutant STAT1-F77A, Y701F with additionally disrupted N domain interactions was used (lane 15). We therefore inferred that N domains—but not single SH2:phosphotyrosine interactions—sustained semiphosphorylated heterodimers, supportive of an antiparallel conformation. Fully phosphorylated STAT dimers enter the nucleus bound to importin-α5 [25,26]. To compare importin binding of the semiphosphorylated variant, WT STAT1 or the nonphosphorylatable STAT1-Y701F (both YFP-tagged) were coexpressed with FLAG-tagged importin-α5, and STAT activation was induced by IFN-ẞ treatment as before (Fig 4B). It should be noted that although experimental IFN-β-induced semiphosphorylated heterodimers (Y701F-STAT1:STAT2-P) and their natural IFN-γ-induced counterparts (P-STAT1:STAT2-U) are not identical, they are both defective in carrier-mediated nuclear import [27], which suggests that they can be used interchangeably to study importin binding. As shown in Fig 4B, importin-α5 coprecipitated with both endogenous and heterologous STAT1 and STAT2, but only in extracts from IFN-ẞ-treated cells (lanes 1 and 2 for STAT1; and 7 and 8 for STAT2), which agrees with the requirement of STAT activation for importin-α5 binding [24].

Semiphosphorylated STAT dimers, in contrast, did not bind importin-α5, as demonstrated by the lack of STAT1-Y701F coprecipitation with importin-α5 (lane 4), despite abundant phosphorylated STAT2 in the extract (lane 22). These results predicted that acquisition of IFN-γ responsiveness by STAT2 abrogated its inhibition of STAT1 nuclear import. This was tested by swapping the SH2 domain from STAT1 into STAT2, which led to the IFN-γ-inducible tyrosine phosphorylation of the hybrid STAT2 reported by Darnell’s group [28]. Before IFN treatment, the hybrid STAT2, like WT, accumulated in the cytoplasm together with STAT1 (Fig 4C, panels 1 and 2). Unlike WT STAT2, but as predicted, hybrid STAT2 did not inhibit the nuclear import of activated STAT1, which accumulated strongly in the nucleus upon IFN-γ treatment (Fig 4C, panels 3 and 4). Restoration of IFN-γ unresponsiveness of hybrid STAT2 by introducing the SH2 domain-inactivating mutation R602L [29], on the other hand, restored STAT1 inhibition (Fig 4C, panels 5 and 6). In conclusion, the inhibition of activated STAT1 required heterodimerization specifically with unphosphorylated STAT2, because such dimers adopted an antiparallel conformation incompatible with binding DNA and importin-α5.

### STAT2 Suppresses IFN-γ-Induced Gene Expression

**Fig 5:**
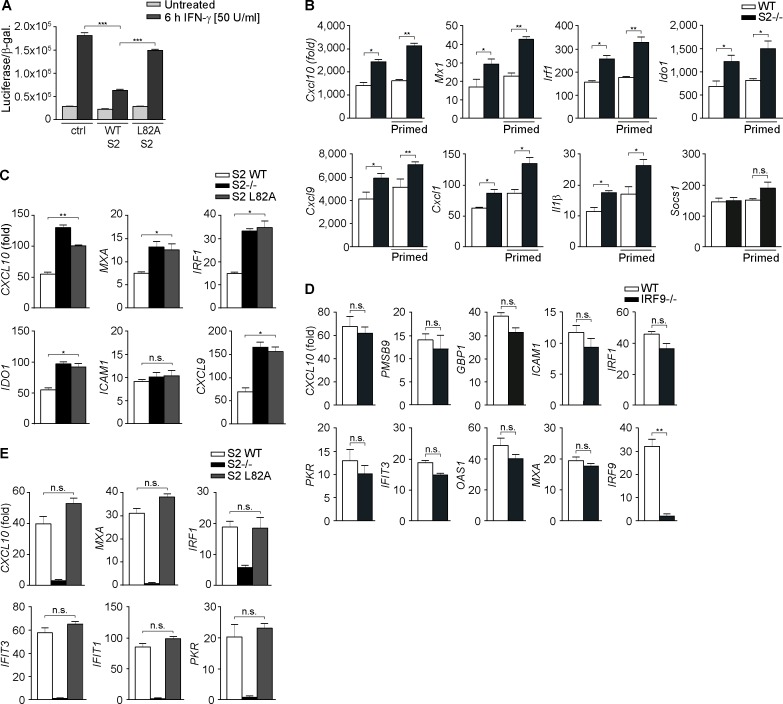
Role of IRF9 and STAT2:STAT1 heterodimerization for gene induction by IFN-γ and IFN-β. **(A-D)** Gene expression results in response to IFN-γ. **(A)** STAT1-responsive (3xGAS) luciferase-reporter gene assay with YFP-tagged WT STAT2 (WT S2) or mutant L82A (L82A S2) or empty expression vector (ctrl). Bars show mean and standard error of the mean (s.e.m.) of six independent β-galactosidase-normalized experiments. **(B-E)** Quantitative RT-PCR analyses with immortalized mouse macrophages **(B)**; parental (S2-/-) and stably STAT2-reconstituted (S2 WT or S2 L82A) human U6A cells **(C)**; and IRF9-deficient U2A (IRF9-/-) and parental 2fTGH cells (WT) **(D).** Where indicated, cells were pretreated (primed) with IFN-γ (1 U/ml) for 48 h. **(E)** identical to (C) except that treatment was with IFN-β. * *p* < 0.05, ** *p* < 0.01 and *** *p* < 0.001. Bars show mean and s.d. of 2–3 independent experiments. See S1 Data for raw data.

We then evaluated the consequences for IFN-γ-induced gene transcription. STAT2 overexpression in HeLa cells resulted in significantly reduced STAT1 reporter gene activity (Fig 5A). The mutant STAT2-L82A, in contrast, displayed significantly higher reporter gene activity (Fig 5A). To elucidate the role of STAT2 for the transcription of endogenous IFN-γ-regulated genes, we used quantitative RT-PCR and immortalized WT and STAT2-deficient mouse macrophages, whereby a similar picture emerged. Aside from notable exceptions such as the IFN negative feedback regulators *Socs1* and *Isg15*, most IFN-γ-regulated genes tested showed significantly increased gene expression in the absence of STAT2, including *CxCl9*, *CxCl10*, *Irf1*, *Ido1*, *Il1β*, and *Marco* (Fig 5B; S7A Fig). STAT2-deficient macrophages contained reduced STAT1 protein, which could be normalized to WT levels by IFN-γ priming (S7B Fig), which further accentuated the enhanced IFN-γ-inducible gene expression of the STAT2-deficient cells (Fig 5B). We expanded the analysis to STAT2-deficient and stably STAT2-reconstituted human U6A cells, which confirmed significantly enhanced IFN-γ-inducible gene expression in cells that lacked STAT2 or expressed the STAT1-binding mutant STAT2-L82A compared to WT STAT2-expressing cells (Fig 5C; S7C Fig). In contrast, a previous study reported that a subset of IFN-γ target genes including *PKR*, *IFIT3*, *MXA*, and *OAS1* was actually induced by semiphosphorylated STAT1:STAT2 heterodimers, albeit in association with IRF9 [30]. We used IRF9-deficient U2A cells, which exhibited slightly reduced STAT1 expression and activation compared to the parental 2fTGH cells (S7D Fig), yet found that lack of IRF9 resulted in no significant difference in the expression of nine genes tested, including the aforementioned subset of genes after a 4 h IFN-γ exposure (Fig 5D). We additionally tested the serine phosphorylation of STAT1, which is required for its full-transcriptional activity [3], and found no differences between the WT and mutant STAT2-reconstituted cells (S7E Fig). We moreover assessed gene induction in response to type 1 IFN (Fig 5E; S4A Fig). For the 17 genes tested, we found that transcription was strongly reduced or lost in the absence of STAT2, as expected, but no difference was seen between cells expressing WT and mutant STAT2, in line with undisturbed type 1 IFN-induced activation and nuclear import of STAT1 and STAT2 (Fig 2A; S4B Fig). Thus, the loss of constitutive STAT2 binding to STAT1 enhanced gene expression in response to IFN-γ, but was without overt consequences for type 1 IFN signaling.

### STAT2 Modulates Key IFN-γ Effector Functions In Vitro and In Vivo

**Fig 6:**
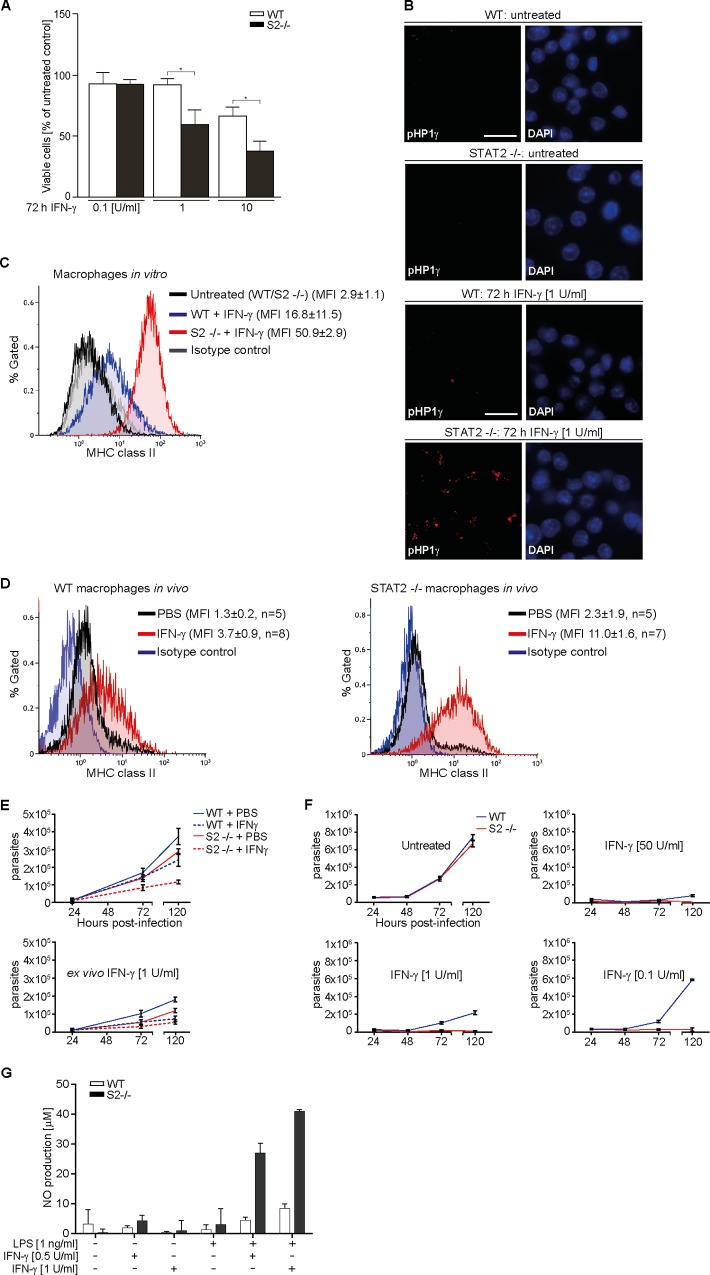
STAT2 modulates key IFN-γ effector functions. **(A)** Viability of immortalized macrophages was measured by Alamar blue assay of mitochondrial function after 72 h in the presence of IFN-γ. **(B)** Widefield microscopy detecting HP1γ (anti-phospho-S83 HP1γ antibody) in immortalized macrophages before (top four panels) and after (bottom four panels) treatment with IFN-γ. Nuclei were DAPI stained. Scale bar = 15 μm. **(C)** Major histocompatibility complex (MHC) II expression on immortalized WT and STAT2-/- macrophages treated without or with 0.1 U/ml IFN-γ for 72 h was determined by fluorescence-assisted cell sorting (FACS) using Alexa Fluor 488-conjugated specific and isotype control antibodies. Gating for live cells was performed using forward and side scatter analysis, and the same gate was used for the histograms in (C) and (D). MFI, median fluorescence intensity. **(D)** Same as (C), but using peritoneal leukocytes from mice injected with PBS or IFN-γ. MHC class II expression (PerCP-conjugated antibody) was analyzed on macrophages detected by Alexa-Fluor 488-conjugated anti-F4/80 antibody. A representative histogram is shown and median PerCP fluorescence intensities ± s.d. per cohort (*n* = animal numbers) are given. **(E)** Peritoneal macrophages from PBS or IFN-γ-injected mice were cultured without (top) or with added IFN-γ (bottom) in duplicates, infected with *Toxoplasma gondii*, and extracellular parasites were counted at the indicated time points. Graphs combine the results from five control and eight experimental mice per genotype, bars show mean and s.d. **(F)** *T*. *gondii* release from infected immortalized macrophages treated with different IFN-γ concentrations. Results represent two independent experiments, bars show mean and s.d. **(G)** Nitric oxide (NO) production by immortalized macrophages determined using Griess reagent in triplicates after 36 h of the indicated treatments, bars show mean and s.d. See S1 Data for raw data.

IFN-γ is a potent cell growth inhibitor and can promote cell death [8]. The influence of STAT2 on different antiproliferative effects of IFN-γ was assessed with immortalized WT and STAT2-deficient macrophages. Using Alamar blue reagent, we found that IFN-γ decreased cell viability of both WT and mutant macrophages in a concentration-dependent manner, although the STAT2-deficient cells were significantly more sensitive and already showed ≈40% decreased viability at a low IFN-γ concentration (1 U/ml) that left WT cells unaffected (Fig 6A). Annexin V staining indicated that this phenomenon entailed apoptosis, as expected. At all IFN-γ concentrations tested, the fraction of apoptotic cells was 2–4 times higher for the STAT2-deficient cells compared to WT (S8A Fig). A particularly noticeable difference between WT and STAT2-deficient macrophages was found when the induction of heterochromatin foci, a marker for cellular senescence, was assessed (Fig 6B; S8B Fig) [31]. The STAT2-deficient cells were labelled strongly with antibody against phosphorylated heterochromatin protein 1-gamma (pHP1γ) even at a low IFN-γ concentration (1 U/ml), indicative of extensive chromatin reorganization characteristic of cellular senescence. WT cells, in contrast, were only weakly responsive to IFN-γ, even at the 50-fold increased concentration (S8B Fig). These experiments documented that lack of STAT2 sensitized cells to multiple growth-inhibitory effects of IFN-γ.

Type 1 and 2 IFNs up-regulate class I and II antigen presentation pathways, whereby IFN-γ alone efficiently induces the class II pathway [8]. Prior to treatment with IFN-γ, major histocompatibility complex (MHC) class II expression was essentially indistinguishable between WT and STAT2-deficient immortalized macrophages (Fig 6C). Treatment of the WT macrophages with IFN-γ increased the surface expression of MHC class II molecules. Yet, while WT cells showed ≈5-fold increase in median fluorescence intensities (MFIs), a ≈15-fold increase was observed with the STAT2-deficient macrophages (Fig 6C), even though a very low IFN-γ concentration was used (0.1 U/ml). Similar, albeit more modest, differences were observed for the induction of MHC class I molecules on human U6A cells (S9A Fig). At the three IFN-γ concentrations tested, STAT2-deficient and mutant STAT2-L82A-reconstituted cells were indistinguishable and up-regulated MHC I significantly better than the WT STAT2-expressing cells. To assess the consequences of STAT2 deficiency for IFN-γ signaling in vivo, we injected WT and STAT2 gene-deleted mice on two consecutive days with IFN-γ or PBS (controls) and collected peritoneal leucocytes 24 h later. Flow cytometry and fluorescent labelling of F4/80 and MHC II molecules were used to identify peritoneal macrophages and to calculate their MFIs in order to compare the effect of IFN-γ injection on MHC II expression. Based on the MFI values, IFN-γ injection of WT mice led to about a 3-fold increased macrophage MHC II expression (Fig 6D, left), whereas cells from the STAT2-deficient animals showed 5-fold increase (Fig 6D, right). In fact, the direct comparison revealed an 8-fold difference between WT and mutant mice, since in the latter MHC II levels were already twice as high under control conditions (Fig 6D). We concluded that STAT2-deficient mice were IFN-γ hyper-responsive and displayed STAT1 gain-of-function phenotype regarding antigen presentation pathways.

The boosting of antimicrobial effector mechanisms is another cardinal IFN-γ-mediated immune response. We therefore performed infection experiments with the obligate intracellular protozoan *T*. *gondii*, whose elimination requires IFN-γ signaling in humans and mice [32]. *T*. *gondii* infection, moreover, does not elicit type 1 IFNs and their potential disease-exacerbating comorbidities [33]; a phenomenon observed in the course of many infections and an important consideration, particularly in the context of STAT2 deficiency and hence disrupted type 1 IFN signaling [34]. At first, WT- and STAT2-deficient mice were injected with IFN-γ or PBS (controls), before peritoneal macrophages were collected for ex vivo infection experiments with *T*. *gondii* tachyzoites (Fig 6E). IFN-γ injection protected both WT and mutant mice-derived macrophages from *T*. *gondii*-induced cell lysis and concomitant parasite release into the culture medium, as parasite numbers were about halved (Fig 6E, top panel). Yet, the STAT2-deficient mice were protected better. Parasite numbers with macrophages from the PBS-injected mutant mice already were about 20% lower compared to WT, and this difference increased to about 50% with the cells from IFN-γ-injected mice (Fig 6E, top panel). The addition of IFN-γ to the ex vivo macrophage cultures reduced *T*. *gondii* propagation further (Fig 6E, bottom panel), whereby again cells from the STAT2-deficient mice were more resistant to the parasite. Together, these experiments confirmed that STAT2 deficiency was associated with the gain-of-function phenotype for another key IFN-γ activity in vivo, namely cell-autonomous antiparasitic immunity. To corroborate these findings, we treated immortalized WT and STAT2-deficient macrophages for two days with a low (“priming”) IFN-γ concentration (1 U/ml) to induce anti-*T*. *gondii* activity, before infection and continued cultivation without or with varying IFN-γ concentrations (Fig 6F). In the IFN-γ-untreated cells, parasite release into the culture medium became apparent ≈48 h post infection; and over the following three days extracellular parasite numbers increased about 10-fold, irrespective of STAT2 expression (Fig 6F, upper left panel). At the highest concentration used, IFN-γ protected both cell lines against parasite propagation almost completely (Fig 6F, upper right panel). The same high level of protection was conferred on STAT2-deficient cells even at the 50- and 500-fold IFN-γ dilutions tested (Fig 6F, lower panels). The WT cells, however, gradually lost the ability to control parasite growth with decreasing IFN-γ concentrations (lower panels). Additional infection experiments were performed with STAT2-deficient and reconstituted human U6A cells. While in this cell line IFN-γ conferred considerably weaker protection against *T*. *gondii*, STAT2-deficient and mutant STAT2-L82A reconstituted cells were indistinguishable and suppressed parasite propagation significantly better than WT STAT2-expressing cells, in line with a suppressive effect of STAT2 on STAT1 and IFN-γ (S9B Fig). Finally, the production of cytotoxic nitric oxide (NO) was assessed by treating cells either with IFN-γ or bacterial lipopolysaccharide (LPS) alone or combined, which resulted in synergistic NO up-regulation both in WT and STAT2-deficient macrophages (Fig 6G) [35]. However, the STAT2-deficient cells released 4–5 times more NO than WT at both IFN-γ concentrations tested (Fig 6G). Taken together, these experiments demonstrated that STAT2 dampens key immunomodulatory activities of IFN-γ both in vitro and in living mice.

### STAT2 Balances the Contributions of STAT1 and STAT3 to IL-27 Transcription Output

**Fig 7:**
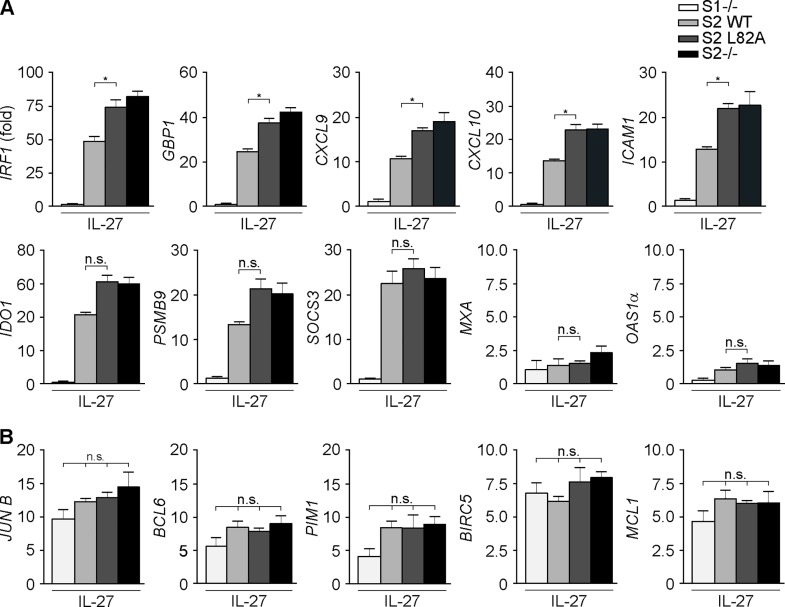
STAT2 shifts the transcriptional output of IL-27 from STAT1 to STAT3. **(A, B)** Gene expression analyses using quantitative reverse transcription polymerase chain reaction (qRT-PCR) with human U3A (S1-/-) cells and parental (S2-/-) and stably STAT2-reconstituted (S2 WT or S2 L82A) human U6A cells treated for 4 h with 100 ng/ml IL-27. * *p* < 0.05, bars show mean and s.d. of 2–3 independent experiments. See S1 Data for raw data.

IL-27 signals via the concomitant activation of both STAT1 and STAT3, but only STAT1 nuclear translocation was inhibited by STAT2 (Fig 3B). To examine how this affected gene induction, we selected several genes whose expression is known to depend on STAT1 or STAT3, respectively, and compared their induction using IL-27 in STAT2-deficient U6A cells and U6A cells reconstituted with WT STAT2 or the L82A mutant. Ten STAT1-regulated genes were selected based on their responsiveness to IFN-γ [21], while five STAT3 targets were chosen based on their IL-6 responsiveness [36]. Dependency on STAT1 was confirmed by the lack of expression in IL-27-treated U3A cells (Fig 7A), a STAT1-deficient human fibrosarcoma line [28]. The five selected STAT1-independent genes tested (*JUNB*, *BCL6*, *PIM1*, *BIRC5*, and *MCL1*), in contrast, were expressed equally well in WT cells and the STAT1-deficient U3A cells (Fig 7B), presumably due to the activation of STAT3. There was, moreover, no significant influence of STAT2 on the IL-27-induced expression of this set of genes (Fig 7B). Contrary, of the ten STAT1-dependent genes tested, five (*IRF1*, *GBP1*, *CXCl9*, *CXCl10*, *ICAM1*) showed significantly increased gene expression in response to IL-27 both in the absence of STAT2 and in the presence of the L82A mutant compared to WT STAT2 (Fig 7A), thus resembling the situation with IFN-γ (Fig 5B and 5C). Three additional genes likewise showed increased IL-27 responsiveness in the absence of STAT2, albeit the differences did not reach significance; while the remaining two STAT1-dependent genes tested, *MXA* and *OAS1*, were unresponsive to IL-27. Although the reason for their unresponsiveness is presently unknown, a contribution of STAT2 was ruled out, as they did not gain responsiveness in the absence of STAT2 (Fig 7A). We additionally explored a possible alternative explanation for the differential IL-27 responsiveness of genes in the cells expressing WT or mutant STAT2, namely differences in STAT1 tyrosine and serine phosphorylation, but found comparable phosphorylation levels in both cell types using western blotting (Fig 3A, S7E Fig). Collectively, these data indicate that STAT2 is a general STAT1 inhibitor whose activities include balancing the contributions of STAT1 and STAT3 to the transcription output of IL-27.

## Discussion

STAT2 is a founding member of the STAT family of transcription factors [37]. It is considered a STAT protein with an uncharacteristically narrow activity profile, namely as a facilitator solely of type 1/3 IFN signaling [13]. Here, we identify STAT2 as a pervasive cytokine regulator due to its inhibition of STAT1 in multiple signaling pathways. The results represent a substantial expansion of known STAT2 biological roles, beyond type 1/3 IFN signaling, and firmly in the IFN-γ, IL-27, and IL-6 pathways. However, it is the unphosphorylated STAT2 that functions as a STAT1 inhibitor. To our knowledge, this is the first example of a latent STAT protein interfering with the cytokine-inducible activities of another, namely STAT1. STAT1 is traditionally associated with IFN-γ signaling, but functions downstream of many additional cytokine receptors often in conjunction with STAT3. Examples include IL-6, IL-21, and IL-27, the latter of which activates STAT1 and STAT3 strongly and with comparable potency in many cell types. Our analysis of IL-27 signaling has only covered two classes of genes. A first class comprises genes that are unresponsive in the absence of STAT1, i.e., whose expression is regulated solely by STAT1. For many of these, STAT2-mediated reduction in nuclear STAT1 translates directly in diminished transcription, similar to the effects on IFN-γ. In contrast, STAT1 does not appear to make a contribution at all for a second class of genes examined, i.e., genes that are induced similarly well in the absence and presence of STAT1. In agreement with STAT2 inhibition being specific for STAT1, but not STAT3, their expression is insensitive to the presence of STAT2. The situation likely is more complex for a sizable third class of genes not examined here where expression is coregulated by STAT1 and STAT3 or other STATs [12]. For these genes, STAT2-mediated inhibition of STAT1 is expected to shift promoter occupancy away from STAT1 to STAT3 and possibly other STATs with difficult-to-predict consequences for the IL-27 transcriptome. While this and other questions await clarification, the already acquired data identify STAT2 as a crucial component of a filtering mechanism for IL-27 and other cytokines whose biological outcomes critically depend upon balancing the transcriptional potency of STAT1 and competing STAT proteins [12,38].

Our results showed that only STAT1 molecules free of STAT2 are available for mediating cytokine functions, such that the modulation of the STAT1:STAT2 protein ratio provides a mechanism for cells to fine tune their cytokine responses and possibly contributing to cell type specificity if the distinctions become permanent. Our limited survey of cell lines has demonstrated that STAT1 is present in excess over STAT2, and that the STAT1:STAT2 ratio of these IFN-stimulated genes was increased further by treatment with IFNs. In this manner, the exposure of cells to IFNs contributes to their increased IFN-γ responsiveness and will tweak the biological activities of multiple additional cytokines towards the STAT1 component in their transcription output. The identification of the STAT1:STAT2 protein ratio as a key determinant for the functioning of STAT1 moreover invalidates the assumption that STAT1 tyrosine phosphorylation can be equated with its biological activity. This is an important consideration, as cytokine responses are dynamic and depend on the strength of the incoming stimulus. IFN-γ-mediated antibacterial immunity, for example, can be viewed as a continuous quantitative trait, where cell activation correlates with outcome of infection [39]. We therefore suggest taking STAT2 protein expression into account when assessing STAT1 bioactivity, including as predictor of prognosis in cancer and disease development [40]. Heterodimers of STAT1 and STAT2 are well known to bind the transcription factor IRF9. This can occur both before and after their type 1 IFN-induced phosphorylation, resulting in trimeric transcription inducers termed U-ISGF3 and ISGF3, respectively [3,41]. A similar interaction between IRF9 and semiphosphorylated STAT1:STAT2 heterodimers was previously shown to promote IFN-γ signaling [30]. In light of our findings, this suggests that IRF9 may alleviate the inhibitory activity of such heterodimers. Our assessment of immediate early and hence direct transcription responses, however, did not provide evidence for this, although we cannot formally rule out that IRF9 contributes to late transcriptional responses involving semiphosphorylated STAT1:STAT2 heterodimers, possibly by an indirect mechanism. Irrespective, semiphosphorylated dimers of both STAT1 and STAT3 resulting from experimental and clinical tyrosine-phosphorylation mutations are associated with defective nuclear import and dominant-negative consequences for cytokine signaling [42–44]. This includes the demonstration that nonphosphorylatable STAT1 inhibits the nuclear import of activated STAT2 as well as gene induction in response to type 1 IFNs [27], making this artificial system the exact complement of what we find in the natural setting with IFN-γ.

The loss of STAT2 can be described as a gain-of-function phenotype for STAT1, of which unrestrained IFN-γ activity is an important consequence, as shown here. However, previous studies with STAT2-deficient patients and mice failed to reveal this aspect. This is probably due to at least three reasons: firstly, although increased IFN-γ responses such as gene induction were noticed occasionally [14], these observations were not pursued as the focus of those studies was on type 1 IFN rather than IFN-γ. Secondly, those studies concentrated on the tyrosine phosphorylation of STAT1, which not only is unaffected by STAT2 but moreover an insufficient measure for the biological activity of STAT1 and IFN-γ, as we have discussed above. Thirdly, and more importantly, STAT1 protein concentrations are reduced by 50%–80% in STAT2-deficient cells from both patients and mice compared to WT [14,15]. This phenomenon, which is probably caused by the disruption of a STAT2-dependent IFN-ẞ feedback loop [45], may abolish the STAT1 gain-of-function phenotype associated with STAT2 deficiency. The prolonged exposure to IFN-γ, however, for example during chronic or acute inflammation, can increase STAT1 protein to WT levels such that the gain-of-function phenotype can come into effect and may manifest as disease exacerbation such as seen for LPS-induced murine sepsis [17]. In conjunction with the considerable functional overlap of type 1 and 2 IFNs, on the other hand, compensatory outcomes can ensue, as suggested by the capacity of IFN-γ to induce an antiviral state in the absence of type 1 IFN signaling [46] and contribute to the unexpectedly narrow role for STAT2 deficiency in human antiviral immunity [15]. Further work is required to verify these possibilities, which will be facilitated by the STAT1-binding mutant described in this work, as it allows the dissociation of STAT2’s effects on type 1 and type 2 IFN. As such, N domain-mediated STAT2 heterodimerization moreover is a potentially attractive target for small molecule-based enhancement of endogenous IFN-γ activity.

The identification of STAT2 as another perpetual STAT1 inhibitor, in addition to SUMO [47], underscores the importance of constitutive signal-dampening mechanisms in STAT1 biology to avoid disease associated with STAT1 hyperactivity [48,49]. However, STAT2 does not target the activation of STAT1, but the subsequent step, its nuclear import. This is a new cytokine modulation mechanism for a STAT protein, but one reminiscent of viral IFN evasion strategies, e.g., by Ebola virus VP24 protein [50]. The results moreover provide an alternative interpretation for the functioning of the STAT1ẞ splice variant, which in lacking a transactivation domain is generally considered a STAT1 antagonist with an unclear biological role [51]. In light of the findings reported here, it can be seen as a STAT2 quencher–through its N domain–and hence rather as a promoter of cytokines that signal via STAT1. Finally, the constitutive interactions between STAT2 and STAT1 are exceedingly tight, yet highly vulnerable to mutation of either STAT1 (F77A) or STAT2 (L82A), suggesting strong evolutionary pressure in favor of heterodimerization. Counterintuitively, heterodimerization was dispensable for the assembly and the functioning of canonical ISGF3 (this work and [21]). It thus appears that the binding of STAT2 to STAT1 exists not because it supports the cytokine inducible activities of STAT2, but because it attenuates those of STAT1.

## Materials and Methods

### Ethics Statement

Procedures were undertaken in accordance with the UK Animals Scientific Procedures Act under project license 40/3601.

### IFN Treatment of Mice

C57BL6/6J mice were purchased from Charles River UK; B6.129-Stat2tm1Shnd/J (Stat2-/-) mice [14] were obtained from Dr. Thomas Decker, Universität Wien, with the permission of Dr. Christian Schindler, Columbia University. Eight to ten wk-old males with a mean body weight of 25 g (21 g–29 g) were injected with 5 μg of carrier-free mouse IFN-γ (2.0–10.0 x 10^6^ units/mg, Biolegend #575308) on two consecutive days. On day three the mice, being in apparently healthy state, were sacrificed by cervical dislocation followed by peritoneal lavage [52] and tissue collection. Lavage samples for FACS analysis were fixed for 30 min in 4% PFA in PBS at 4°C prior to antibody staining. All mice that entered the study are included in the final analysis described in the figures with the exception of one: A STAT2 -/- mouse, injected with IFN-γ, was found to have an exceptionally high MHC class II expression on peritoneal macrophages (MFI 33.6). Whilst this followed the general trend of macrophages from STAT2 -/- animals having greater MHC class II up-regulation in response to IFN-γ, this animal was treated as an outlier and subsequently not included in calculating the average median intensity for the relevant cohort.

### Cell Culture and Transfections

Immortalized bone marrow-derived macrophages from WT and STAT2 -/- mice were obtained from authors of [17] and grown as described. U6A cells were stably reconstituted with YFP-tagged human STAT2 or STAT2-L82A and isolated by FACS. U2A, U3A, and U6A cell lines were obtained from G. Stark [53]. See S1 Text for additional details.

### Cytokines and Other Treatments

Human (#407306) and mouse (#407320) IFN-γ, human IL-6 (#407652) were purchased from Merck Millipore, human IFN-β (#11415–1) was from PBL Assay Science. Human IL-6Rα was from R&D Systems (#227-58-025), human IL-27 from Peprotech (#200–38). Other treatments used in this work include LPS (#L7895, Sigma) and ratjadone (#553590, Merck Millipore). Unless stated otherwise, IFN treatment was for one hour with 50 U/ml IFN-γ or 500 U/ml IFN-β in growth medium. Similarly, unless stated otherwise, IL-27 treatment was performed for 40 min at a concentration of 100 ng/ml. IL-6/IL-6R cotreatments also lasted for 40 min at concentrations of 200 and 250 ng/ml, respectively.

### Experiments with *T*. *gondii*

*T*. *gondii*, provided by Dr. Gereon Schares, were grown and purified as previously described [54]. Briefly, tachyzoites were maintained in vitro by serial passage through Madin-Darby Canine Kidney (MDCK) cells in DMEM supplemented with 5% FBS, 2 mM glutamine, and 1% (v/v) antibiotic-antimycotic solution (Gibco). Immediately prior to infection, tachyzoites were purified from their feeder cell cultures by passage through PD-10 desalting columns. The purified tachyzoites were centrifuged at 1,000 × g, and the parasite pellet was suspended in fresh culture medium. Parasites were counted with an improved Neubauer hemocytometer. Peritoneal macrophages for ex vivo *T*. *gondii* experiments were isolated as previously described [55]. Briefly, lavage cells were plated onto Poly-L-lysine coated glass coverslips and incubated for 3 h in DMEM supplemented with antibiotics and 10% FBS (growth medium). Nonadherent cells were removed by rigorous washes with PBS and the remaining adherent macrophages were fed fresh growth medium. Lavage cells were plated at a density of 1 x 10^5^ cells per well with intention to obtain a density of 5 x 10^4^ peritoneal macrophages per well. Actual densities achieved were determined for each mouse by counting an additional coverslip used subsequently to calculate MOI. Immortalized macrophages and U6A cells were plated on poly-L-lysine-coated 24-well culture plates (Nunc) at a density of 5 x 10^4^ cells per well and incubated in 0.5 ml growth medium for 15 h. Cells were left untreated or treated for 48 h with IFN-γ at the indicated concentrations, before infection for 5 h with tachyzoites at a MOI of 3. Unattached parasites were washed away with PBS, and cells were fed fresh growth medium containing the same IFN-γ concentration as before. Extracellular parasites were counted using 100 μl medium samples, followed by replacement with the appropriate culture medium such that IFN-γ concentrations were maintained. Data from at least two biological replicates were evaluated, and averages were calculated.

### NO Assay and Alamar Blue Assay

Determination of NO and cell viability, using Griess reagent (Promega) and Alamar blue assay (Bio-Rad), respectively, was done as described by the manufacturers. See S1 Text for additional details.

### FACS to Detect MHC Expression and Cell Death

For MHC expression analyses, cells were grown for 72 h in the absence or presence of IFN-γ as indicated. Apoptosis analyses were done with cells left untreated or treated for 48 h with IFN-γ as indicated. Following treatment, cells were detached and suspended in ice-cold PBS containing 0.2% heat-inactivated mouse serum (MS). After two washes in this buffer by centrifugation at 300 x g for 5 min each, cell suspensions (≈2.5 x 10^5^ cells in 100 μl) were incubated for 20 min at RT with 4 μg/ml Alexa Fluor-488 rat antimouse IA/I-E (#107615, Biolegend) or 4 μg/ml Alexa Fluor-488 rat IgG2b, κ isotype control (#400625, Biolegend) for the detection of MHC class II expression on mouse macrophages. MHC class I on human U6A cell lines was detected by the same procedure using 3.2 μg/ml APC-conjugated anti-human HLA-A,B,C (#311409, Biolegend) or APC-conjugated mouse IgG2a, κ isotype control (#400219, Biolegend). For analysis of peritoneal lavage samples, PFA-fixed cell suspensions were incubated for at least 30 min in PBS containing 0.2% heat-inactivated MS prior to 20 min incubation at RT with 5 μg/ml Alexa Fluor-488 anti-mouse F4/80 (#123119, Biolegend), 2 μg/ml PE/Cy7 anti-mouse CD3 (#100219, Biolegend), 2 μg/ml PE anti-mouse CD45R/B220 (#103207, Biolegend) and 4 μg/ml PerCP anti-mouse IA/I-E (#107623, Biolegend). Isotype controls were carried out using identical concentrations of Alexa Fluor-488 rat IgG2a, κ (#400525, Biolegend), PE/Cy7 IgG2b, κ (#400617, Biolegend), PE rat IgG2a, κ (#400507, Biolegend) and PerCP IgG2b, κ (#400629, Biolegend) control antibodies. F4/80+ cells were identified as peritoneal macrophages and their MHC class II expression was subsequently analyzed. Apoptosis and necrosis were analyzed by simultaneous detection of cell surface annexin V and propidium iodide as described by the manufacturer (#640914, Biolegend), whereby Biolegend’s cell staining buffer was replaced with MS. Immediately before FACS using FC500 (Beckman-Coulter) or LSRII flow cytometers (Beckman-Coulter), cell suspensions were passed once through a 20G needle on a 1 ml syringe. FACS data were processed using Kaluza analysis software (Beckman Coulter, version 1.3).

### Plasmids and Molecular Cloning

See S1 Text for additional details.

### Gene Expression Analyses

Quantitative reverse transcription polymerase chain reaction (qRT-PCR) was done as described [21]. See S1 Text for additional details.

### Fluorescence Microscopy

Endogenous proteins were detected after fixation of cells for 15 min in ice-cold methanol and blocking for 1 h in 20% (v/v) FBS/PBS prior to 15 h incubation at 4°C with primary antibodies. A Zeiss Axioplan 2 microscope and AxioVision 4.7 software (Zeiss) were used for widefield immunofluorescence imaging; deconvolution microscopy was done with a DeltaVision Elite microscope (GE Lifesciences) and Resolve 3D software (Softworx). Line scan analysis of STAT protein subcellular distribution was performed using ImageJ. Within each cell the YFP, CFP, or Cy3 fluorescence intensity within the cytoplasm or nucleus was measured across separate 10.6 μm line scans. The MeFI across the cytoplasmic line scan was divided by that of the nucleus; producing a ratio of cytoplasmic:nuclear fluorescence intensity for the cell. Data shown represent at least 25 cells per condition. See S1 Text for additional details.

### Quantitative Western Blotting

Whole cell extractions, SDS-PAGE, and quantitative western blotting were as described [56]. Results combine or are representative of at least two independent experiments. See S1 Text for additional details.

### Immunoprecipitation and EMSA

For STAT1 precipitation experiments, HEK 293T cells were cotransfected with expression vectors for STAT2 and FLAG-tagged STAT1 variant proteins, or pCMV-FLAG-N expression vector as control. For importin-α5 precipitations, FLAG-tagged importin-α5 [50] and vector encoding YFP-tagged STAT1 or STAT1-Y701F was cotransfected, with pEGFP-N1 used as control. The cells were treated without or with IFN-β for 1 h before extraction. Extracts were incubated with anti-FLAG M2 magnetic beads (Sigma). For EMSA, cell extracts were normalized for Tyr701-phosphorylated STAT1 and incubated with radiolabeled GAS (M67) or ISRE (ISG15) probes as described [21]. See S1 Text for additional details.

### Protein Preparation and Analytical Ultracentrifugation

STAT N domain expression in insect cells, affinity-purification (Strep-Tag), and sedimentation analyses were as described [22]. Sedimentation equilibrium data were obtained with STAT1 N domain and mEGFP-tagged STAT2 N domain at final equimolar concentrations of 2.5, 10, and 40 μM. See S1 Text for additional details.

### Statistical Analyses and Software

Statistical significance was calculated by Student’s *t* test (* denotes *p* < 0.05; ** *p* < 0.01; and *** *p* < 0.001; n. s. denotes *p* > 0.05) performed using GraphPad Prism 5.03 (GraphPad). Quantitative western blot analysis was performed using Image Studio Lite (Li-Cor, version 3.1).

## Supporting Information

S1 Fig STAT2 overexpression does not trigger cytoplasmic redistribution of STAT3 and STAT4. HeLa cells transiently overexpressing YFP-tagged STAT2 were fixed and stained with antibodies detecting endogenous STAT3 and STAT4 and a Cy3-coupled secondary antibody as indicated. Nuclei were DAPI-stained. Scale bar = 15 μm.
(TIF)

S2 Fig NES-mediated nuclear export of STAT2 drives the accumulation of STAT1 in the cytoplasm. **(A)** STAT1-CFP and YFP-tagged C-terminally-deleted STAT2 (STAT2-ΔC) were expressed in HeLa cells and their subcellular localization was observed by deconvolution microscopy. Scale bar = 15 μm. **(B)** HeLa cells co-expressing STAT1-CFP and STAT2-YFP were treated with 10 ng/ml ratjadone for 6 h before fixation and viewing as in (A). Scale bar = 15 μm.
(TIF)

S3 Fig STAT2 single mutant L82A and double mutant LL81,82AA are functionally identical. STAT1-CFP and the indicated YFP-tagged STAT2 wild type or mutant variants were co-expressed in HeLa cells and their subcellular localization was observed by deconvolution microscopy. Scale bar = 10 μm.
(TIF)

S4 Fig Abrogated heterodimerization between STAT1 and STAT2 does not affect their IFN-β-induced nuclear import and gene induction. **(A)** Gene induction determined by qRT-PCR with parental (S2-/-) and stably STAT2-reconstituted (S2 WT or S2 L82A) human U6A cells after treatment with IFN-β (500 U/ml) for 4 h. **(B) **STAT1-CFP and YFP-tagged wild type (top panels) or L82A mutated STAT2 (bottom panels) were co-expressed in HeLa cells and their subcellular localization was observed by deconvolution microscopy after 1 h in the presence of IFN-β. Note assembly and co-localization of STAT1 and WT STAT2, but not the mutant STAT2, in nuclear bodies, presumably paracrystals. Such structures and their loss upon the disruption of N domain-mediated (antiparallel) homodimerization have been demonstrated for WT STAT1 and mutant F77A [57]. Scale bar = 10 μm. See S1 Data for raw data.
(TIF)

S5 Fig Effects of WT and mutant STAT2 on IFN-mediated STAT1 activation. Parental (S2-/-) and stably STAT2-reconstituted (S2 WT or S2 L82A) human U6A cells were left untreated (-) or treated (+) with IFN as indicated. Western blotting results with whole cell extracts are shown using antibodies (denoted by α) detecting phospho-Y701 STAT1 (pS1), STAT1 (S1), phospho-Y690 STAT2 (pS2), STAT2 (S2), and β-actin. kD, kilo Dalton.
(TIF)

S6 Fig Determination of the relative binding affinities of STAT1 and STAT2 antibodies. **(A)** Whole cell extracts of HEK 293T cells co-expressing STAT1-YFP and STAT2-YFP were serially diluted as indicated, resolved by SDS-PAGE and probed with anti-GFP antibody (lanes 1–6) and re-probed with either anti-STAT1 (lanes 7–12, bottom) or anti-STAT2 (lanes 7–12, top). GFP expression (lane 1) was used as antibody control. **(B)** The bar graph combines the results of (A) with a technical replicate. Given are the mean numerical values of the STAT2-YFP/STAT1-YFP signal ratios determined using anti-GFP (value A; white bars) or the respective anti-STAT1 and anti-STAT2 antibodies (value B; black bars). [B/A] was determined to be 5.7 and is the fold-difference in binding affinity between anti-STAT2 and anti-STAT1. Bars show mean and s.d. See S1 Data for raw data.
(TIF)

S7 Fig Influence of L82A mutated STAT2 and increased STAT1 concentration relative to STAT2 on IFN-γ induced gene induction; assessment of IRF9 expression on the IFN-mediated activation of STAT1 and STAT2; and assessment of cytokine-induced STAT1 serine 727 phosphorylation. **(A) **Gene induction determined by qRT-PCR with immortalized macrophages following treatment with IFN-γ (50 U/ml) for 4 h. Where indicated, cells were pre-treated (primed) with 1 U/ml IFN-γ for 48 h. **(B) **Western blotting experiments showing the effect of 48 h treatment with the indicated concentrations of IFN-γ on STAT1 (αS1) and STAT2 (αS2) expression in macrophages. **(C)** Same as in (A) but with parental (S2-/-) and stably STAT2-reconstituted (S2 WT or S2 L82A) human U6A cells. * *p* < 0.05, ** *p* < 0.01. RT-PCR results are representative of three independent experiments; bars show mean and s.d. **(D) **Western blotting experiments investigating the effect of IRF9 expression on IFN-induced tyrosine phosphorylation of STAT1 and STAT2. Parental 2fTGH cells (WT) and IRF9-deficient U2A cells (IRF9-/-) were left untreated or treated for 1 h with IFN-γ or IFN-β as indicated. Results with whole cell extracts are shown using antibodies (denoted by α) detecting phospho-Y701 STAT1 (pS1), STAT1 (S1), phospho-Y690 STAT2 (pS2), STAT2 (S2), IRF9 and β-actin. kD, kilo Dalton. **(E)** Western blotting experiments investigating the effect of STAT2-L82A on STAT1 serine 727 phosphorylation. U6A cells stably expressing STAT2 or STAT2-L82A were left untreated or treated with IFN-γ (50 U/ml); IL-27 (100 ng/ml); or co-treated with Il-6 (200 ng/ml) and soluble IL-6R (250 ng/ml) for 40 minutes. Results with whole cell extracts are shown using antibodies (denoted by α) detecting phospho-S727 STAT1 (pS1S727), STAT1 (S1) and β-actin. kD, kilo Dalton. See S1 Data for raw data.
(TIF)

S8 Fig STAT2-deficient cells are sensitized to anti-proliferative effects of IFN-γ. **(A)** Flow cytometric analysis of cell death in WT (top) and STAT2-deficient (bottom) macrophage cell lines. Cells were left untreated or treated with increasing concentrations of IFN-γ for 48 h, before analysis of apoptosis and necrosis by simultaneous detection of cell surface annexin V and DNA binding of propidium iodide. **(B)** Widefield immunofluorescence microscopy images showing labelling with anti-pHP1γ antibody (red) in immortalized WT and STAT2-deficient macrophages left untreated or treated with 10 (top) and 50 (bottom) U/ml IFN-γ for 72 h; nuclei are blue. Scale bar = 15 μm.
(TIF)

S9 Fig STAT2 regulates cardinal IFN-γ responses. **(A) **Representative histograms of MHC class I expression on U6A (middle) and WT STAT2- (left) and STAT2-L82A-reconstituted (right) cells. Cells were left untreated or treated for 72 h with 1 (top), 5 (middle) and 50 (bottom) U/ml IFN-γ. Dashed lines denote mode intensity values for untreated and IFN-treated cells, respectively. MFI, median fluorescence intensities. **(B)** Influence of IFN-γ on *T*. *gondii* propagation in parental and stably STAT2-reconstituted human U6A cell. After infection (t = 0), extracellular parasites were counted at the indicated time points using a Neubauer chamber. Results are representative of two independent experiments carried out in duplicates, bars show mean and s.d. See S1 Data for raw data.
(TIF)

S1 Text Supplemental Material and Methods, References. (DOCX)

S1 Data Underlying data and quantitative observations presented in figures. (XLSX)

## Abbreviations

CFP: cyan fluorescent protein
EMSA: electrophoretic mobility shift assay
FACS: fluorescence-assisted cell sorting
GAF: gamma interferon-activated factor
GAS: gamma interferon-activated site
IFN: interferon
IL-6: interleukin-6
IL-27: interleukin-27
IP: immunoblots of precipitates
ISG: IFN-stimulated gene
ISGF3: interferon-stimulated gene factor 3
ISRE: interferon-stimulated response element
JAK: Janus kinase
K_D_: equilibrium dissociation constant
LPS: lipopolysaccharide
MHC: major histocompatibility complex
MeFI: mean fluorescence intensity
MFI: median fluorescence intensity
MS: mouse serum
NES: nuclear export signal
NO: nitric oxide
N domains: aminoterminal domains
pHP1γ: phosphorylated heterochromatin protein 1-gamma
qRT-PCR: quantitative reverse transcription polymerase chain reaction
s.d.: standard deviation
s.e.m.: standard error of the mean
SH2: src kinase homology 2
SOCS: suppressor of cytokine signaling
STAT: signal transducer and activator of transcription
WT: wild type
YFP: yellow fluorescent protein
