# Supplementary figures and images for: STAT2 Is a Pervasive Cytokine Regulator due to Its Inhibition of STAT1 in Multiple Signaling Pathways
Source: PLoS Biol. 2016 Oct 25;14(10):e2000117. doi: 10.1371/journal.pbio.2000117 (PMC5079630; doi:10.1371/journal.pbio.2000117)

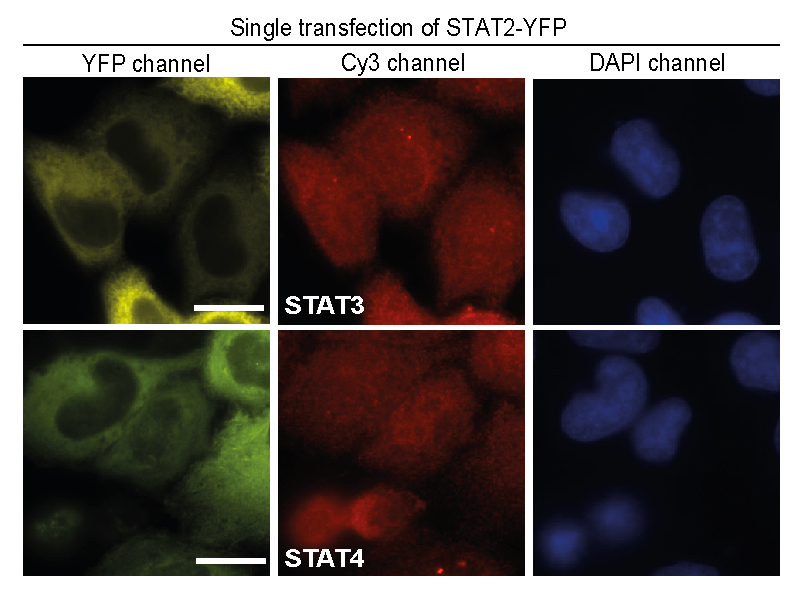

Supplement: S1 Fig — HeLa cells transiently overexpressing YFP-tagged STAT2 were fixed and stained with antibodies detecting endogenous STAT3 and STAT4 and a Cy3-coupled secondary antibody as indicated. Nuclei were DAPI-stained. Scale bar = 15 μm. (TIF) [file pbio.2000117.s001.tif]

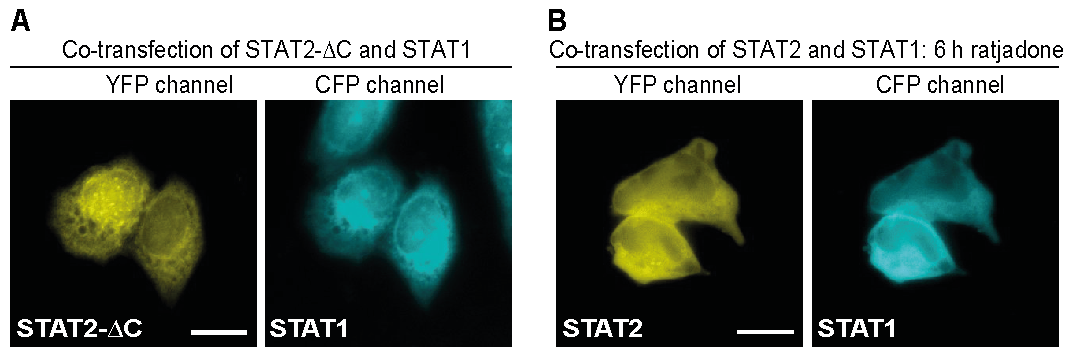

Supplement: S2 Fig — (A) STAT1-CFP and YFP-tagged C-terminally-deleted STAT2 (STAT2-ΔC) were expressed in HeLa cells and their subcellular localization was observed by deconvolution microscopy. Scale bar = 15 μm. (B) HeLa cells co-expressing STAT1-CFP and STAT2-YFP were treated with 10 ng/ml ratjadone for 6 h before fixation and viewing as in (A). Scale bar = 15 μm. (TIF) [file pbio.2000117.s002.tif]

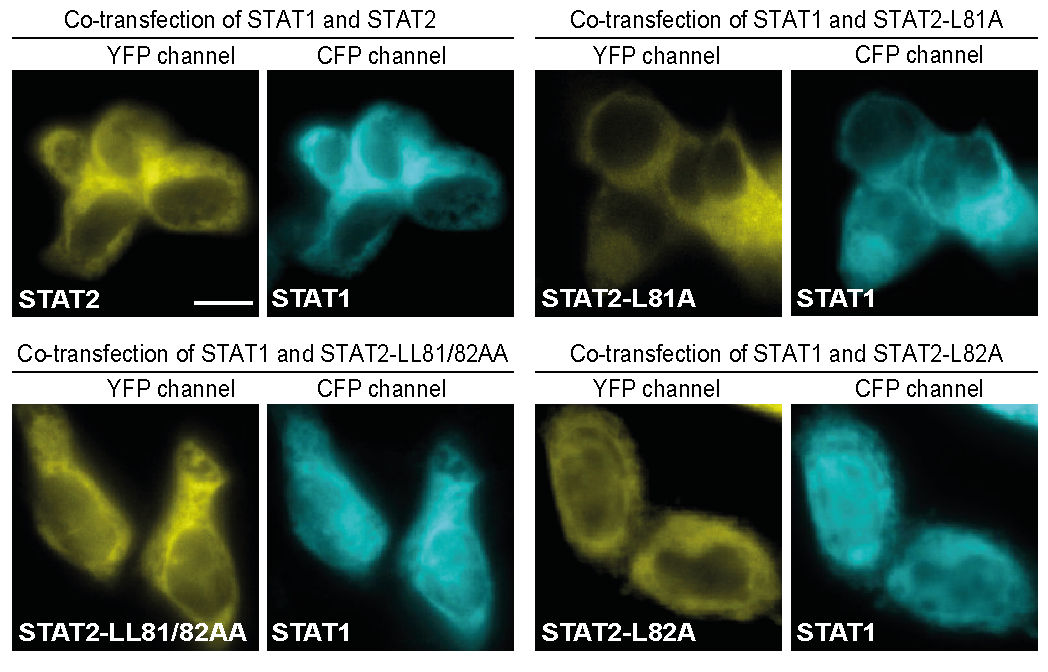

Supplement: S3 Fig — STAT1-CFP and the indicated YFP-tagged STAT2 wild type or mutant variants were co-expressed in HeLa cells and their subcellular localization was observed by deconvolution microscopy. Scale bar = 10 μm. (TIF) [file pbio.2000117.s003.tif]

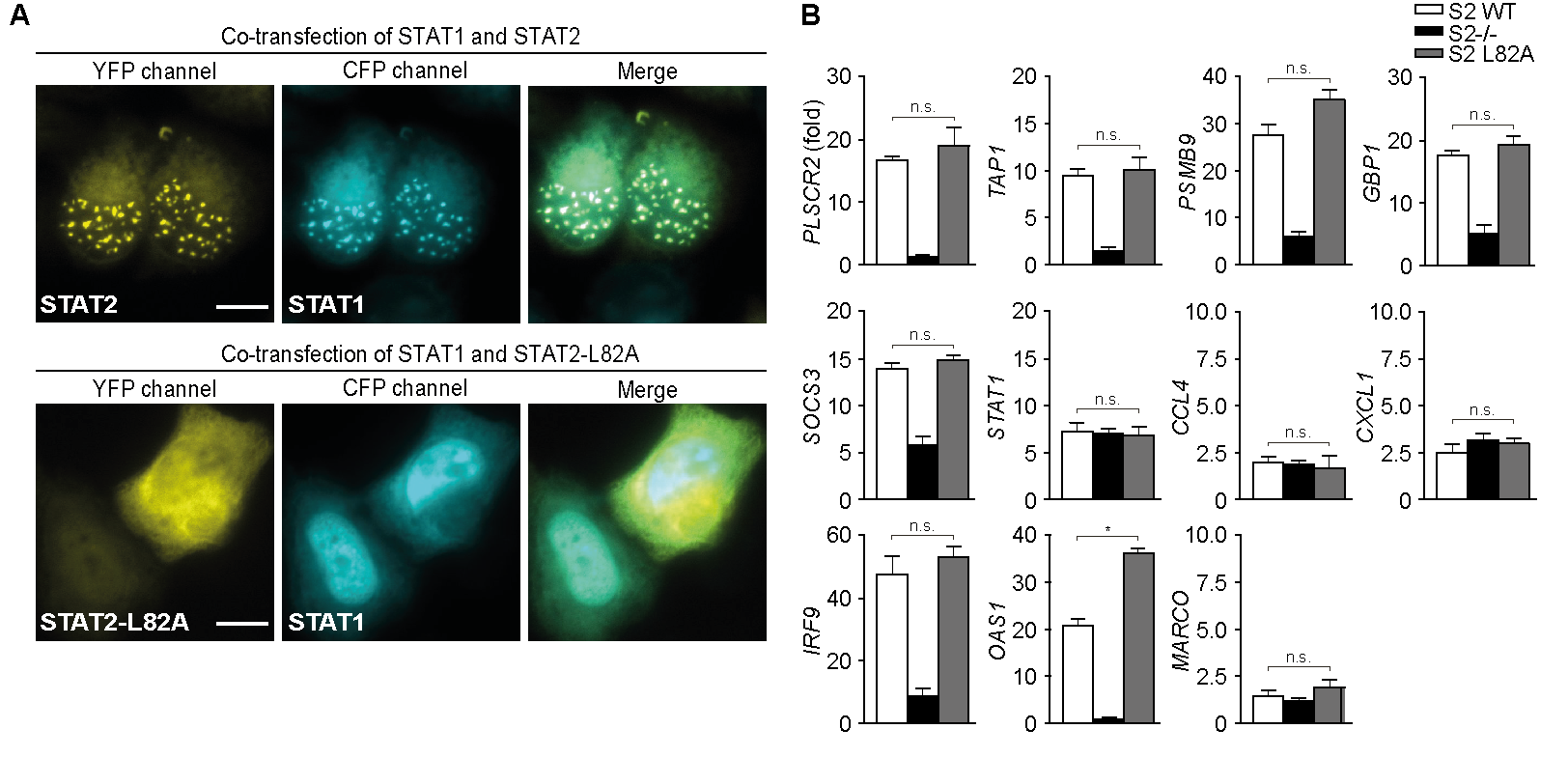

Supplement: S4 Fig — (A) Gene induction determined by qRT-PCR with parental (S2-/-) and stably STAT2-reconstituted (S2 WT or S2 L82A) human U6A cells after treatment with IFN-β (500 U/ml) for 4 h. (B) STAT1-CFP and YFP-tagged wild type (top panels) or L82A mutated STAT2 (bottom panels) were co-expressed in HeLa cells and their subcellular localization was observed by deconvolution microscopy after 1 h in the presence of IFN-β. Note assembly and co-localization of STAT1 and WT STAT2, but not the mutant STAT2, in nuclear bodies, presumably paracrystals. Such structures and their loss upon the disruption of N domain-mediated (antiparallel) homodimerization have been demonstrated for WT STAT1 and mutant F77A [57]. Scale bar = 10 μm. See S1 Data for raw data. (TIF) [file pbio.2000117.s004.tif]

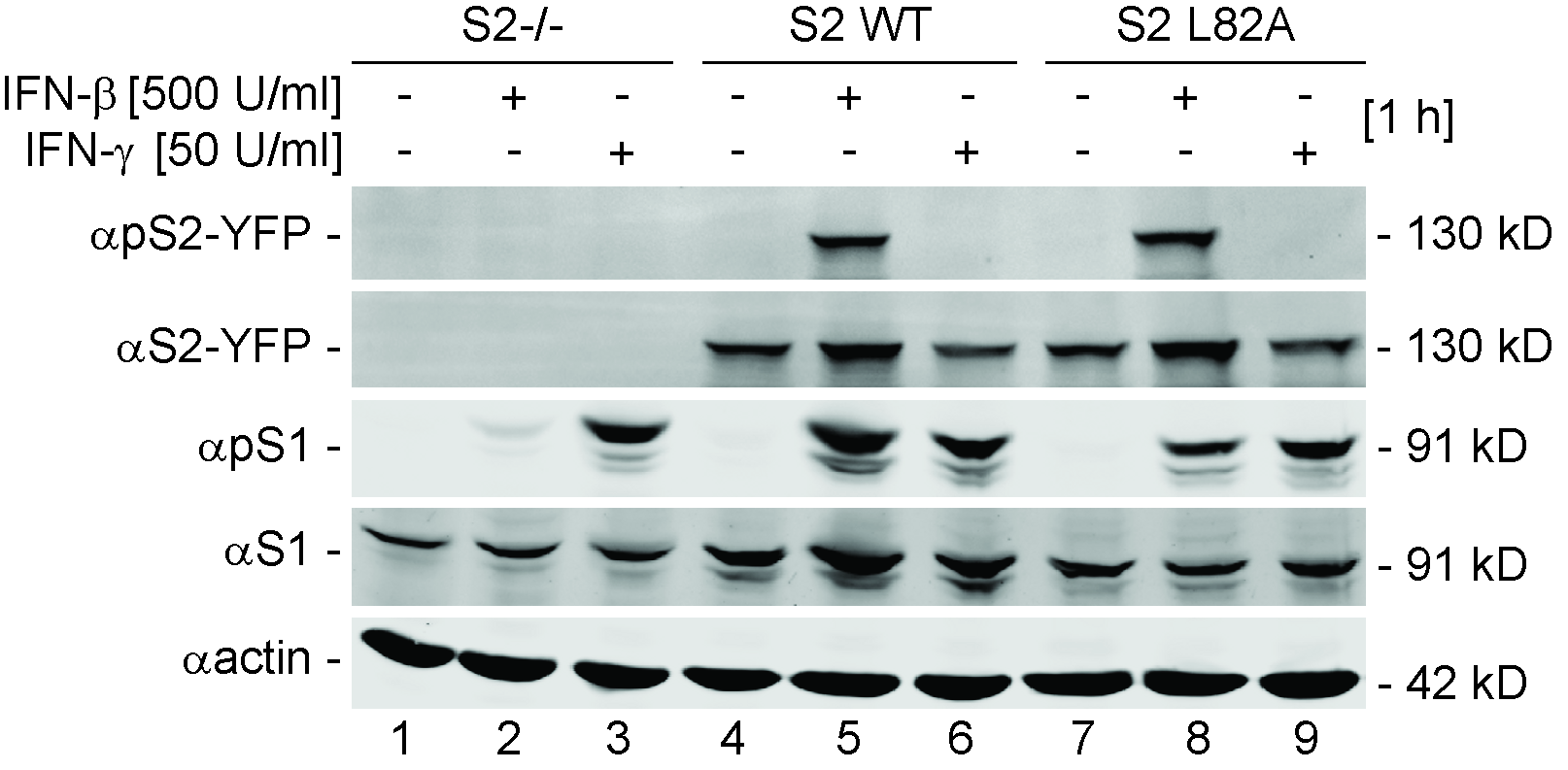

Supplement: S5 Fig — Parental (S2-/-) and stably STAT2-reconstituted (S2 WT or S2 L82A) human U6A cells were left untreated (-) or treated (+) with IFN as indicated. Western blotting results with whole cell extracts are shown using antibodies (denoted by α) detecting phospho-Y701 STAT1 (pS1), STAT1 (S1), phospho-Y690 STAT2 (pS2), STAT2 (S2), and β-actin. kD, kilo Dalton. (TIF) [file pbio.2000117.s005.tif]

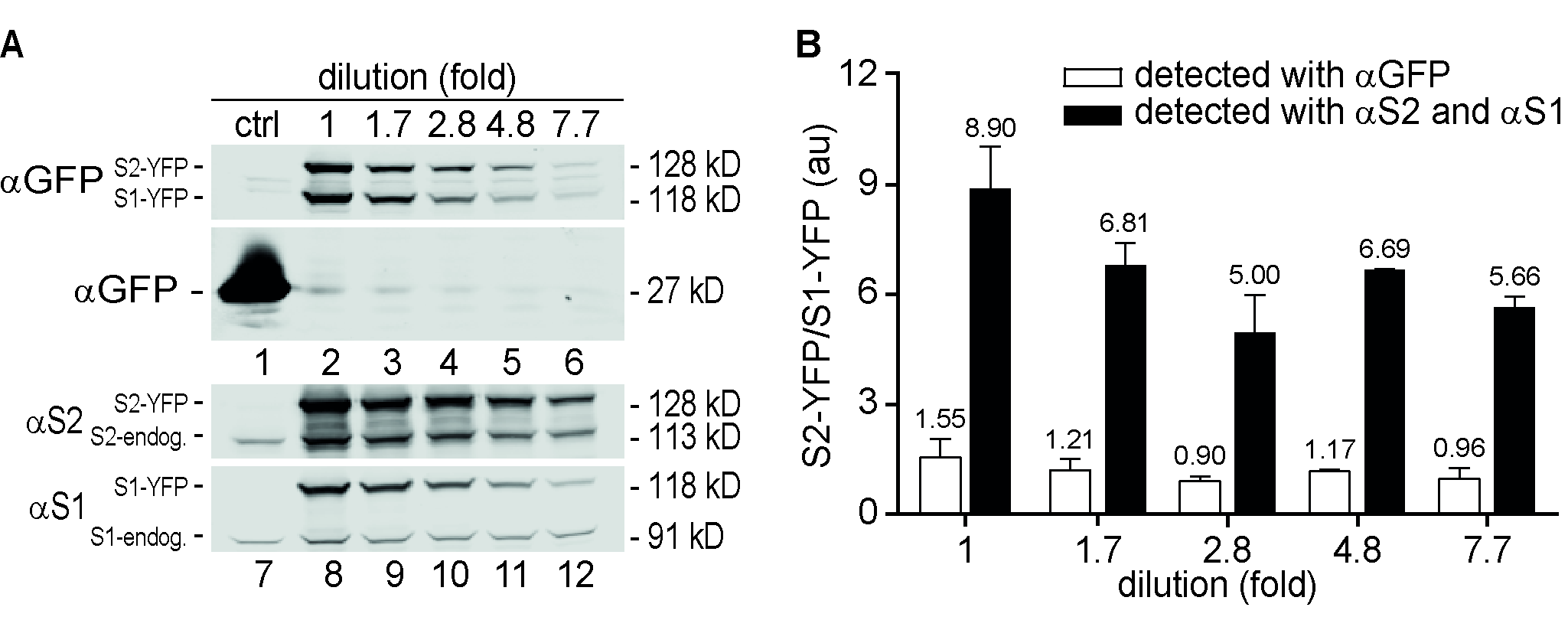

Supplement: S6 Fig — (A) Whole cell extracts of HEK 293T cells co-expressing STAT1-YFP and STAT2-YFP were serially diluted as indicated, resolved by SDS-PAGE and probed with anti-GFP antibody (lanes 1–6) and re-probed with either anti-STAT1 (lanes 7–12, bottom) or anti-STAT2 (lanes 7–12, top). GFP expression (lane 1) was used as antibody control. (B) The bar graph combines the results of (A) with a technical replicate. Given are the mean numerical values of the STAT2-YFP/STAT1-YFP signal ratios determined using anti-GFP (value A; white bars) or the respective anti-STAT1 and anti-STAT2 antibodies (value B; black bars). [B/A] was determined to be 5.7 and is the fold-difference in binding affinity between anti-STAT2 and anti-STAT1. Bars show mean and s.d. See S1 Data for raw data. (TIF) [file pbio.2000117.s006.tif]

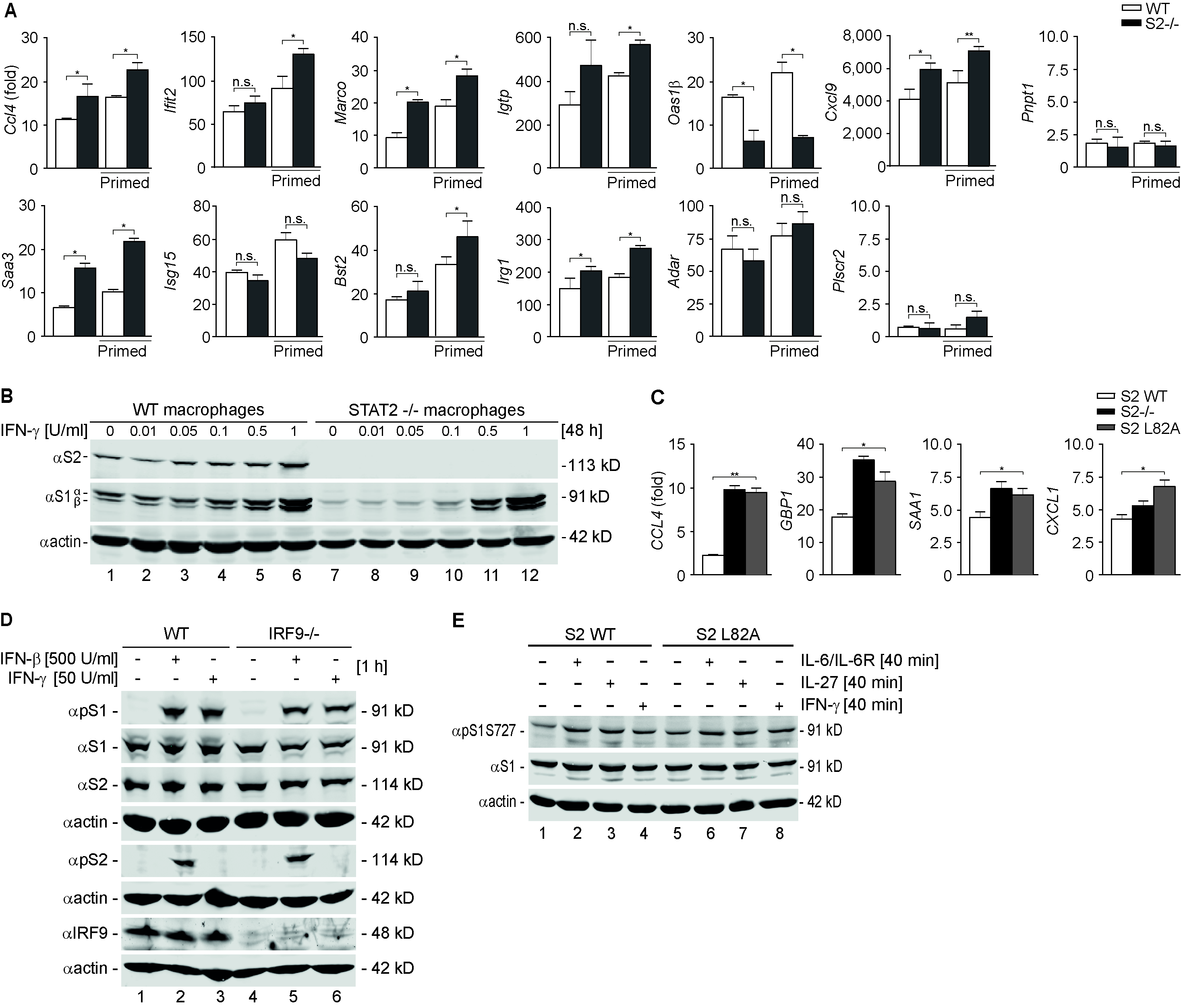

Supplement: S7 Fig — (A) Gene induction determined by qRT-PCR with immortalized macrophages following treatment with IFN-γ (50 U/ml) for 4 h. Where indicated, cells were pre-treated (primed) with 1 U/ml IFN-γ for 48 h. (B) Western blotting experiments showing the effect of 48 h treatment with the indicated concentrations of IFN-γ on STAT1 (αS1) and STAT2 (αS2) expression in macrophages. (C) Same as in (A) but with parental (S2-/-) and stably STAT2-reconstituted (S2 WT or S2 L82A) human U6A cells. * p < 0.05, ** p < 0.01. RT-PCR results are representative of three independent experiments; bars show mean and s.d. (D) Western blotting experiments investigating the effect of IRF9 expression on IFN-induced tyrosine phosphorylation of STAT1 and STAT2. Parental 2fTGH cells (WT) and IRF9-deficient U2A cells (IRF9-/-) were left untreated or treated for 1 h with IFN-γ or IFN-β as indicated. Results with whole cell extracts are shown using antibodies (denoted by α) detecting phospho-Y701 STAT1 (pS1), STAT1 (S1), phospho-Y690 STAT2 (pS2), STAT2 (S2), IRF9 and β-actin. kD, kilo Dalton. (E) Western blotting experiments investigating the effect of STAT2-L82A on STAT1 serine 727 phosphorylation. U6A cells stably expressing STAT2 or STAT2-L82A were left untreated or treated with IFN-γ (50 U/ml); IL-27 (100 ng/ml); or co-treated with Il-6 (200 ng/ml) and soluble IL-6R (250 ng/ml) for 40 minutes. Results with whole cell extracts are shown using antibodies (denoted by α) detecting phospho-S727 STAT1 (pS1S727), STAT1 (S1) and β-actin. kD, kilo Dalton. See S1 Data for raw data. (TIF) [file pbio.2000117.s007.tif]

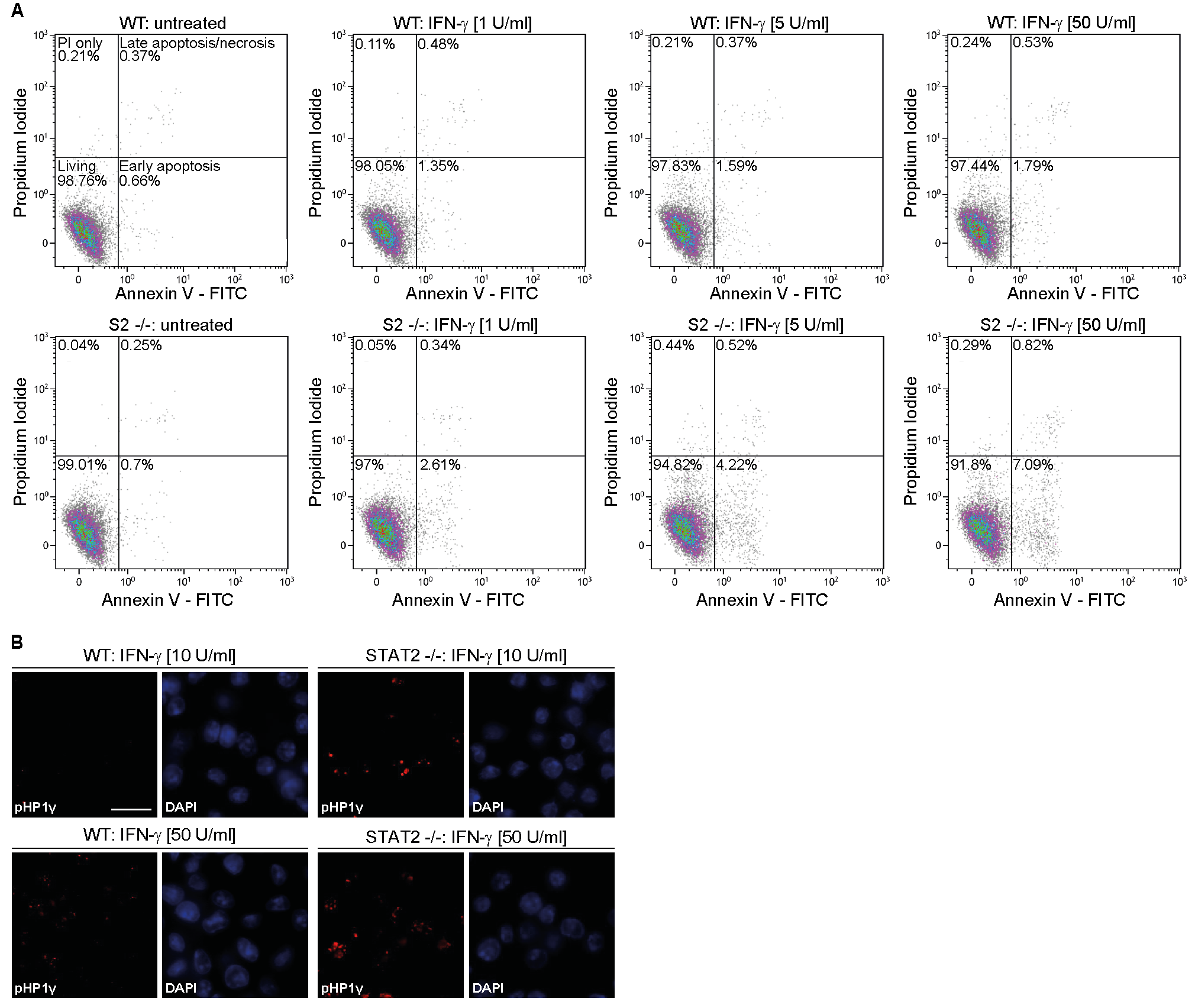

Supplement: S8 Fig — (A) Flow cytometric analysis of cell death in WT (top) and STAT2-deficient (bottom) macrophage cell lines. Cells were left untreated or treated with increasing concentrations of IFN-γ for 48 h, before analysis of apoptosis and necrosis by simultaneous detection of cell surface annexin V and DNA binding of propidium iodide. (B) Widefield immunofluorescence microscopy images showing labelling with anti-pHP1γ antibody (red) in immortalized WT and STAT2-deficient macrophages left untreated or treated with 10 (top) and 50 (bottom) U/ml IFN-γ for 72 h; nuclei are blue. Scale bar = 15 μm. (TIF) [file pbio.2000117.s008.tif]

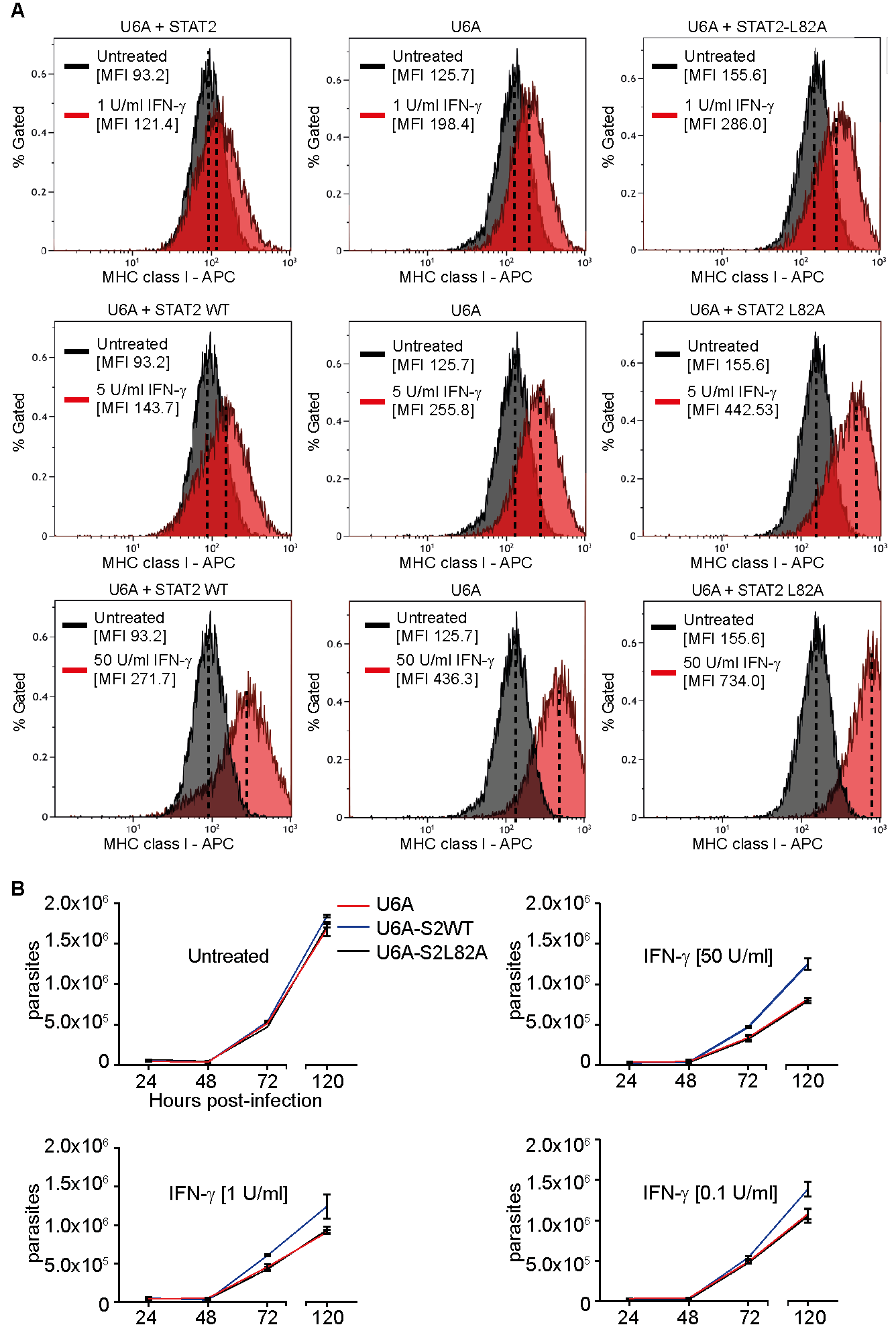

Supplement: S9 Fig — (A) Representative histograms of MHC class I expression on U6A (middle) and WT STAT2- (left) and STAT2-L82A-reconstituted (right) cells. Cells were left untreated or treated for 72 h with 1 (top), 5 (middle) and 50 (bottom) U/ml IFN-γ. Dashed lines denote mode intensity values for untreated and IFN-treated cells, respectively. MFI, median fluorescence intensities. (B) Influence of IFN-γ on T. gondii propagation in parental and stably STAT2-reconstituted human U6A cell. After infection (t = 0), extracellular parasites were counted at the indicated time points using a Neubauer chamber. Results are representative of two independent experiments carried out in duplicates, bars show mean and s.d. See S1 Data for raw data. (TIF) [file pbio.2000117.s009.tif]
