# Supplementary material for: STAT2 Is a Pervasive Cytokine Regulator due to Its Inhibition of STAT1 in Multiple Signaling Pathways
Source: PLoS Biol. 2016 Oct 25;14(10):e2000117. doi: 10.1371/journal.pbio.2000117 (PMC5079630; doi:10.1371/journal.pbio.2000117)
Supplement: S1 Text — (DOCX) [file pbio.2000117.s010.docx]

**Supplemental Materials and Methods, References**

**Cell culture and transfections**

Immortalized bone marrow-derived macrophages from WT and STAT2 -/- mice were provided by Drs. William Alazawi and Ana Gamero [17]. HEK 293T and U2OS cells were provided by Dr. Rob Layfield (University of Nottingham). Sf9 insect cells were provided by Dr. Claire Friel (University of Nottingham). RAMOS and BJAB cell lines were provided by Dr. Ian Daniels (Nottingham University Hospitals Trust, NHS). HeLaS3 were obtained from Deutsche Sammlung von Mikroorganismen und Zellkulturen GmbH. HepG2 (HB-8065) and PC-3 (CRL-1435) cell lines were obtained from ATCC. All cell lines (except Sf9) were grown in humidified incubators at 37°C with 5% CO2. Cells (except BJAB, RAMOS, Sf9) were cultured in growth medium, that is Dulbecco’s Modified Essential Medium (DMEM, Sigma) supplemented with 1% (v/v) penicillin/streptomycin (Sigma) and 10% (v/v) heat-inactivated fetal bovine serum (FBS, Sigma). RAMOS and BJAB cells were grown in RPMI 1640 (Sigma) medium supplemented with 1% (v/v) penicillin/streptomycin and 10% or 20% (v/v) FBS, respectively. Sf9 cells were cultured at 27°C in Insect-XPRESS™ medium (Lonza) supplemented with 1% (v/v) penicillin/streptomycin, 1% (v/v) Pluronic F-172 (Thermo Fisher) and Amphotericin B (Sigma). Incubation at room temperature (RT) in Trypsin/EDTA solution (Sigma) was used to detach adherent cells during experiments and passage. Transient transfection of cells was performed as described [56]. U6A cells were reconstituted with human STAT2 or STAT2-L82A in a pEYFP-N1 expression vector. Cells were transfected as described [56] and cultured in growth medium containing 400 µg/ml G418 (Thermo Fisher) for 14 days; STAT2 positive cells were then isolated using FACS by gating for yellow fluorescent protein (MoFlo cell sorter, Beckman Coulter).

**Nitric oxide assay**

Immortalized mouse macrophages were primed in growth medium containing 0.5 U/ml IFN-γ for 48 h. Cells were subsequently washed with PBS, detached and seeded onto 96-well plates at a density of 1 x 10^4^ cells/well. Cells were incubated for further 36 h in growth medium without or with IFN-γ and LPS as indicated. NO production was assessed by nitrite determination using Griess assay (Promega) as described [47].

**Alamar blue assay for cell viability**

Immortalized mouse macrophages on 48-well plates were grown in triplicates for 72 h with or without IFN-γ at the indicated concentrations. Cell cultures and the cell-free control were then incubated for 4 h with 10-fold diluted Alamar Blue (Bio-Rad) as per manufacturer recommendations. Samples were measured at absorbance of 570 nm and 600 nm using a SpectraMax 340PC plate reader (Molecular Devices). The difference in dye reduction between IFN-treated and untreated control cells indicative of proliferation was calculated with software available from Bio-Rad at www.adserotec.com/alamarblue.

**Gene expression analyses**

Luciferase reporter gene assays were performed as previously described [56] with the following modifications. HeLaS3 cells were transfected on 24-well plates with 0.2 μg/well ẞ-galactosidase encoding plasmid, 0.3 μg/well 3xLy6E-luciferase reporter gene, and 0.25 μg/well pEYFPN1-STAT2 or pEYFPN1-STAT2-LL81,82AA; pEYFP-N1 (Clontech Takara) was used as the control. Quantitative reverse transcription polymerase chain reaction (qRT-PCR) was done as described with the following modifications [21]. Cells were kept in serum-reduced (1%) growth medium for 15 h before change to growth medium. After 2 h, IFN-γ (50 U/ml) was added for 4 h. Macrophages were primed with IFN-γ (1 U/ml) for 48 h, the last 15 h of which cells were kept in serum-reduced growth medium. After another 2 h in growth medium, IFN-γ (50 U/ml) was added for 4 h. For mouse genes the following primer pairs in addition to those described in [21] were used. Cxcl1 5’-cgaagtcatagccacactcaa-3’ and 5’-gagcagtctgtcttctttctcc-3’ (120bp product, 60°C annealing temperature), Pnpt1 5’-gggtcatcttctatggcatctg-3’ and 5’-aaccaatcccactgctacac-3’ (105bp, 60°C), Oas1β 5’-aacatcctcaagaagcctaacc-3’ and 5’-gatcgataacttgccctccttc-3’ (106bp, 58°C), Saa3 5’-gcatcttgatcctgggagtt-3’ and 5’-agctttcttcatgtcagagtagg-3’ (107bp, 58°C), Irg1 5’-gtatcattcggaggagcaagag-3’ and 5’-ggaggtgttggaactgtagatt-3’ (122bp, 60°C), Marco 5’-cacagaagacagagccgattt-3’ and 5’-cttcttgggcactggatcat-3’ (82bp, 60°C), Plscr2 5’-cccacattccagactaactacc-3’ and 5’-aagccgtgtagcagcataa-3’ (112bp, 60°C), Ifit2 5’-gagtttgaggacagggtgttta-3’ and 5’-agacctctgcagtgctttac-3’ (100bp, 58°C), Bst2 5’-tgaagtcacgaagctgaacc-3’ and 5’-tgacactttgagcaccagtag-3’ (123bp, 60°C), Adar 5’-cgtcccatgaacctcgattta-3’ and 5’-gtcctgcttggctactttct-3’ (94bp, 58°C), Ccl4 5’-gccctctctctcctcttgctcgt-3’ and 5’-gctgccgggaggtgtaagagaaa-3’ (109bp, 58°C), Il1β 5’-cctcgtgctgtcggacccatatg-3’ and 5’-cagggtgggtgtgccgtctttc-3’ (190bp, 60°C) and Ido1 5’-cccctgggtccttgtggctagaa-3’ and 5’-cggggcagcacctttcgaaca-3’ (204bp, 60°C). For human genes the following primer pairs were used. GAPDH 5’-caatggggaaggtgaaggtcggagtc-3’ and 5’-cagccttgacggtgccatggaatt-3’ (189bp, 60°C), CXCL1 5’-cgaagtcatagccacactcaa-3’ and 5’-gatttgtcactgttcagcatctt-3’ (102bp, 60°C), GBP1 5’-tcgccctggccgtcctcctgag-3’ and 5’-cggggtccagctcttcatcttgtagt-3’ (435bp, 58°C), CXCL9 5’-tggtgttcttttcctcttgggcatcatc-3’ and 5’-ctcgcaggaagggcttggggca-3’ (166bp, 64°C), CXCL10 5’-tgcgattctgatttgctgccttatcttt-3’ and 5’-attcttgatggccttcgattctggattc-3’ (241bp, 60°C), SAA1 5’-tcggctcagacaaatacttcc-3’ and 5’-ggcatcgctgatcacttct-3’ (95bp, 60°C), ICAM1 5’-cggccccctaccagctccagac-3’ and 5’-ggcgccggaaagctgtagatggtcac-3’ (328bp, 60°C), IRF1 5’-atgagaccctggctagag-3’ and 5’-aagcatccggtacactcg-3’ (349bp, 54°C), MXA 5’-ctggtgctgaaactgaagaaac-3’ and 5’-tacctctgaagcatccgaaatc-3’ (99bp, 60°C), CCL4 5’-cctcgcaactttgtggtaga-3’ and 5’-gttccaggtcatacacgtactc-3’ (142bp, 60°C), IDO1 5’-cccttcaagtgtttcaccaaatc-3’ and 5’-gtcttcccagaacccttcatac-3’ (136bp, 60°C), SOCS3 5’-caacgtggccactcttca-3’ and 5’-gctgggtgactttctcatagg-5’ (77bp, 60°C), MCL1 5’-gtctcgagtgatgatccatgtt-3’ and 5’-tggttcgatgcagctttct-3’ (130bp, 56°C), BCL6 5’-catgtacacatctcggctcaa-3’ and 5’-ctctgcttcactggccttaata-3’ (127bp, 56°C), JUNB 5’-accaagagcgcatcaaagt-3’ and 5’-ttgagcgtcttcaccttgtc-3’ (118bp, 56°C), PIM1 5’-gaagtggtcctgctgaagaa-3’ and 5’-ttccgtgatgaagtcgaagag-3’ (116bp, 56°C) and BIRC5 5’-ccaccgcatctctacattca-3’ and 5’-ccaagtctggctcgttctc-3’.

**Plasmids and molecular cloning**

CFP-tagged human STAT1 and STAT1-F77A and YFP-tagged human STAT1 constructs have been described [57]. Plasmid encoding human STAT2 C-terminally fused to YFP (pEYFPN1-STAT2) was provided by Dr. Jacob Piehler, Universität Osnabrück. Deletion of bases encoding aa 703 to 851 of human STAT2 to produce pEYFPN1-STAT2∆C was performed using the Q5 site-directed mutagenesis kit and primers 5’-atggtgagcaagggcgag-3’ and 5’-cacctgtctattagagaccacaatg-3’ as described by the manufacturer (NEB). Side-directed mutagenesis was done with Quik-change II kit (Agilent). pEYFPN1-STAT2-LL81,82AA was generated from pEYFPN1-STAT2 in two consecutive steps using the following primer pairs, for L81A 5’-ccaggacccagagtccgcgttgctgcagcac-3’ and 5’-gtgctgcagcaacgcggactctgggtcctgg-3’; for L82A 5’-gacccagagtccgcggcgctgcagcacaatttg-3’ and 5’-caaattgtgctgcagcgccgcggactctgggtc-3’. Subsequent experiments revealed that mutation L81A was dispensable for abrogation of N domain interactions, thus A81L was introduced in pEYFPN1-STAT2LL81,82AA using primer pair 5’-gacccagagtccttggcgctgcagcacaatttg-3’ and 5’-caaattgtgctgcagcgccaaggactctgggtc-3’. To produce untagged STAT2, a stop codon was introduced at the 3’ end of the STAT2-encoding sequence in pEYFPN1-STAT2 using primers 5’-ttgatgccttctgactaggccggaattctgcag-3’ and 5’-ctgcagaattccggcctagtcagaaggcatcaa-3’. STAT1-FLAG expression construct has been described [58]. It was used to generate (a) STAT1-F77A with primers 5’-ctaaaggaactggatttatcaagactgagt-3’ and 5’-actcagtcttgaaaatccagttcctttag-3’, (b) STAT1-Y701F with primers 5’-ggaactggatttatcaagact-3’ and 5’-agtcttgataaatccagttcc-3’, (c) STAT1-F77A,Y701F by sequential mutagenesis using the primer pairs described. pCMV-FLAG-N expression vector was from Clontech. SH2 domain hybrid STAT2 was generated using splicing by overlap extension (SOE) PCR essentially as described [28] with three oligonucleotide pairs generating PCR fragments encoding aa 1-571 of STAT2 (i), aa 573-710 of STAT1 (ii), and aa 701-851 of STAT2 (iii). The primer pairs used to produce the three fragments were (i) 5’-atatataagcttcggaccatggcgcagtgggaaatg-3’ (A) and 5’-gcacccatcattccagagatccttcaggtggtcatg-3’(B), (ii) 5’-cacctgaaggatctctggaatgatgggtgcatcatg-3’ (C) and 5’-atccacctgtctattagacacagaaatcaactcagt-3’ (D), (iii) 5’-ttgatttctgtgtctaatagacaggtggatgaactg-3’ (E), and 5’-atatatgaattcggaagtcagaaggcatcaagggtcc-3’ (F) (restriction sites underlined). Fragments (i) and (ii) were then subject to SOE PCR to generate fragment (iv) using primers A and D to fuse cDNAs encoding STAT2 aa 1-571 and STAT1 aa 573-701. PCR fragments (ii) and (iii) were fused using primers C and F, encoding STAT1 aa 573-710 and STAT2 aa 701-851 (fragment v). A final SOE PCR with fragments (iv) and (v) using primers A and F was performed. The resulting PCR fragment was cloned into pEYFPN1. Mutation R602L was introduced with primer pair 5’-gggaccttcctgctgctgttcagtgagagc-3’ and 5’-gctctcactgaacagcagcaggaaggtccc-3’. Human STAT1 N domain was produced as described [22]. Human STAT2 N domain (aa 1-126) and STAT2-LL81,82AA (mutagenesis as described before) were produced in baculovirus-infected insect cells essentially as described [22]. To facilitate analyses of STAT heterodimerization, we produced STAT2 N domain fused C-terminally with EGFP. The weak intrinsic dimerization activity of EGFP was further reduced by mutation A206K [59], using primers 5’-ctgagcacccagtccaagctgagcaaagacccc-3’ and 5’-ggggtctttgctcagcttggactgggtgctcag-3’ to generate monomerized EGFP, denoted mEGFP. All N domain constructs expressed a 13 aa linker (LEVDLQGDHGLSA) followed by the Strep-II-tag (WSHPQFEK-stop) at their C-terminus. Cloning details for N domain expression constructs are available upon request. Cloning enzymes were from NEB. DNA fragments were resolved by low melting point agarose (Melford Laboratories) gel electrophoresis and purified with Nucleospin Extract II kit (Macherey-Nagel). DNA amplification was performed by transformation of NEB10β super-competent bacteria (NEB); plasmid DNA was isolated using PureLink Maxiprep columns (Invitrogen). Cloning results were verified by DNA sequencing.

**Fluorescence microscopy**

Cells grown on sterile glass coverslips were washed once with PBS and fixed for 15 min in ice-cold methanol. For YFP/CFP co-transfection experiments, cells were washed twice with PBS following fixation and mounting onto slides using FluorPreserve reagent (Merck Millipore). For labelling of endogenous proteins, cells were fixed for 15 min in ice-cold methanol and then blocked for 1 h in 20% (v/v) FBS/PBS prior to 15 h incubation at 4°C with primary antibodies as follows: 0.4 µg/ml anti-STAT2 (#sc-476, Santa Cruz), 0.2 µg/ml anti-STAT1 (#sc-345, Santa Cruz), 4 µl/ml anti-STAT1 (phospho-Tyr701) (#7649, Cell Signaling), 0.4 µg/ml anti-STAT3 (#sc-482, Santa Cruz), 1:100 diluted anti-STAT3 (phospho-Tyr705) (#9145, Cell Signaling), 1 ug/ml anti-STAT4 (#660502, Biolegend) or 5 µg/ml anti-HP1γ (phospho-Ser93) (#ab45270, Abcam). After three washes in PBS, cells were incubated for 1 h at RT with 0.75 µg/ml of the appropriate species-specific secondary immunoglobulins conjugated to Cy3 (anti-mouse, #115-165-062 JIR, Stratech, or anti-rabbit #A10520, Thermo Fisher Scientific), in 20% (v/v) FBS/PBS. Following two further PBS washes, nuclei were counterstained with 5 µg/ml Hoechst 33258 or 1 µg/ml DAPI (both from Sigma) for 5 min and coverslips mounted onto slides as described above. Widefield indirect immunofluorescence detection was performed with a Zeiss Axioplan 2 microscope and Axiocam CCD camera using the following filter settings: DAPI/Hoechst, excitation (ex) 359 nm/emission (em) 461 nm; Cy3 ex 595 nm/em 620 nm; and YFP, ex 520 nm/em 532 nm. Fluorescent imaging was processed using AxioVision 4.7 (Zeiss). Deconvolution microscopy was performed using a DeltaVision Elite microscope (GE Lifesciences) and a CoolSNAP HQ2 CCD camera (Photometrics). The following filter sets were used to detect: CFP, ex 438 nm/em 470 nm; YFP, ex 513 nm/em 559 nm; Cy3, ex 575 nm/em 632 nm; and DAPI/Hoechst, ex 390 nm/em 435 nm.

**Whole cell extraction, SDS-PAGE and quantitative western blotting**

Whole cell extract preparation, SDS-PAGE, and antibodies for western blotting were as described [21, 56]. Additional antibodies used for western blot were anti-STAT1 (phospho-Ser727) (#9177, Cell Signaling), anti-STAT2 (#sc-476, Santa Cruz), anti-STAT2 (phospho-Tyr690) (#07-224, Merck Millipore), anti-STAT3 (#sc-482, Santa Cruz), anti-STAT3 (phospho-Tyr705) (#9145, Cell Signaling) and anti-IRF9 (#sc-496, Santa Cruz). Quantitative evaluation of STAT1:STAT2 ratios in human cell lines was done as follows. Serial dilutions of extracts from HEK 293T cells co-transfected with STAT1-YFP and STAT2-YFP expression plasmids were analyzed by western blotting (S6 Fig), initially with 1 µl/ml anti-GFP (#2555, Cell Signaling) for 1 h at RT, and subsequently for 1 h at RT with either 0.25 µg/ml anti-STAT1 (#610116, lot: 4220737, BD Transduction Laboratories) or 0.2 µg/ml anti-STAT2 (#sc-476, lot: A0314, Santa Cruz). The intensities of the STAT signals relative to the respective YFP signal were compared. It was found that the anti-STAT2 signal was 5.7 times higher than the anti-STAT1 signal for a given YFP concentration. This difference was applied as a correction factor for comparing endogenous STAT1 and STAT2 protein levels.

**Immunoprecipitation of semiphosphorylated STAT heterodimers**

For STAT1 immunoprecipitation experiments, HEK 293T cells were co-transfected with expression vectors for STAT2 and STAT1, or STAT1-Y701F, or STAT1-F77A, or STAT1-F77A, Y701F (all STAT1 variants with a C-terminal FLAG-tag), or pCMV-FLAG-N expression vector. For importin-α5 immunoprecipitation experiments, HEK 293T cells were co-transfected with FLAG-tagged importin-α5 (provided by Dr. Christopher Basler, Mount Sinai Hospital, USA ) and vector encoding YFP-tagged STAT1 or STAT1-Y701F, pEGFP-N1 vector was used as control. Transfected confluent cells on a 10 cm dish were left untreated or treated for 1 h with 500 U/ml IFN-β. Cells were washed with PBS and lysed with 1 ml ice-cold immunoprecipitation buffer (Ipb) consisting of 1 mM EDTA, 20 mM HEPES/NaOH pH 7.4, 150 mM NaCl, 10 mM KCl, 0.1 mM NaVO3, 10% (v/v) glycerol, 1x Complete protease inhibitor cocktail (Roche), 1 mM DTT, 100 µM PMSF and 0.2% (v/v) IGEPAL CA-630 detergent (Sigma). Lysis was achieved mechanically through the use of a Teflon dounce homogenizer. Insoluble protein was removed via centrifugation at 16,000 x g for 4 min. The extracts were tested for the expression of FLAG-tagged proteins by western blotting, and were subsequently normalized for FLAG-tagged protein content by appropriate dilution with Ipb-extracts from untreated HEK 293T cells. Slurry consisting of 50 % (v/v) anti-FLAG M2 magnetic beads (Sigma) was prepared after two Ipb washes. To each precipitation reaction (0.5 mg protein in 1 ml), 40 µl prepared 50 % bead slurry was added. Protein precipitation and elution with SDS-PAGE sample buffer was performed as described by the manufacturer (Sigma). Precipitates and normalized extracts (inputs) were analyzed by western blot.

**Electrophoretic mobility shift assay (EMSA)**

U6A and stably reconstituted cells were left untreated or treated with IFN-β (500 U/ml) or IFN-γ (50 U/ml) for 1 hour. Cytoplasmic and nuclear extracts were prepared and equal volumes were combined for use in EMSA as described [58]. Prior to EMSA, the content of Tyr701-phosphorylated STAT1 in the combined extracts was determined by quantitative western blot and then normalized by the addition of combined extracts from untreated cells of the same genotype. GAS (M67) and ISRE (ISG15) probes (10 nM double stranded DNA with 5’-tgac-3’ overhangs at the 5’ ends) were labelled and analyzed by EMSA as described [21]. The protein composition of IFN-induced DNA:protein complexes was determined by super shift assay using STAT1 (#sc-591, Santa Cruz) and STAT2 (#sc-950, Santa Cruz) antibodies as described [21] (results not shown).

**Protein preparation**

Generation of baculovirus transfer vector through recombination in DH10bac bacteria and transfection of Sf9 insect cells to produce recombinant baculovirus were done as described in the Bac-to-Bac expression system manual (Invitrogen). Sf9 cells at cell density of 2 x 10^6^ cells/ml were infected at MOI of 2. Cells were harvested after 3 days by centrifugation at 500 x g for 15 min and lysed in ice-cold extraction buffer (pH 7) containing 20 mM MES, 100 mM KCl, 10 mM Na2HPO4/NaH2PO4, 1 mM EDTA, 1 mM EGTA and 1x Complete protease inhibitor (Roche). Mechanical lysis was performed with a dounce homogenizer and lysates were cleared by centrifugation at 15,100 x g for 30 min at 4°C. Polyethyleneimine (#P3143, Sigma) was added (0.05%-0.1% (w/v) final) to precipitate nucleic acids. The lysate was cleared by centrifugation (15 min at 15,100 x g at 4°C) and filtration (0.45 µm pore size) before binding to and elution from a Strep-Tactin Sepharose (#2-1201, IBA) column by gravity flow according to the manufacturer’s protocol. Finally, ~10 mg protein was loaded onto a preparative Superose 6 gel filtration column (#17-0537, GE Healthcare) equilibrated in PBS containing 5 mM β-mercaptoethanol (M3148, Sigma). Column fractions containing the protein of interest were pooled and stored as ammonium sulphate precipitates at 4°C. Protein purity was assessed by SDS-PAGE and Coomassie staining and judged to be > 95%.

**Analytical ultracentrifugation (AUC)**

Experiments were performed using a Beckman Optima XL-I analytical ultracentrifuge (Research Complex at Harwell, UK). Sedimentation velocity experiments were performed at 20°C using both absorbance and interference optics. Ammonium sulphate precipitated proteins (~2-5 mg) were dissolved in 15 ml AUC buffer (50 mM Tris, 100 mM NaCl, 1 mM Tris(2-carboxylethyl)phosphine hydrochloride, pH 8) and concentrated to ~200 µl using Amicon ultra centrifugal filter devices (UFC901008, Millipore). Dilution and concentration were repeated another three times; protein concentrations of Strep-tagged N domains were determined by absorption at 280 nm (Nanodrop 2000 photometer) using the following molar extinction coefficients (L mol^−1^ cm^−1^) obtained from their amino acid sequence using an online tool (web.expasy.org). STAT1, 29,450; WT and LL81,82AA mutant STAT2-mEGFP, 48,360. A protein concentration in the range of 0-60 µM in AUC buffer was loaded in 2-channel centerpieces (Beckman Coulter; Indianapolis, USA), and the sedimentation profiles were scanned at a rotor speed of 50,000 rpm for untagged proteins and at 40,000 rpm for mEGFP-tagged proteins. Experimental data were analyzed for protein purity and aggregates by using c(s) distribution analysis as implemented in SEDFIT [60]. Data confirmed the absence of impurities and non-specific aggregation. Untagged and mEGFP-tagged STAT2-N domains behaved essentially identically (data not shown). Sedimentation equilibrium (SE) data were obtained at 4°C using interference optics and scanned every 5 h until equilibrium was reached. N domain samples were prepared in AUC buffer at the following concentrations: STAT1: 9, 18 and 36 µM; STAT2: 0.25, 1.0 and 4.0 mM. STAT1:STAT2-mEGFP and STAT1:STAT2-LL81,82AA-mEGFP were mixed at final equimolar concentrations of 2.5, 10 and 40 µM. Protein samples (130 µL) were loaded in 2-channel centerpieces and SE experiments were performed at three different rotor speeds. Buffer density and viscosity were measured with a density meter DMA 5000M equipped with Lovis 200M viscometer module (Anton-Paar, Switzerland). Experimental data were prepared in SEDFIT and analyzed in SEDPHAT [60]. Data for STAT1 and STAT2 homodimers were analyzed using a “monomer-dimer self-association” model; Data for heterodimers were analyzed using the “self-association with heterodimer of homodimers” model. The K_D_ values were determined with fixed molar mass, which was calculated using ExPASy (http://web.expasy.org/protparam/). The K_D_ of STAT2-mEGFP was corrected for non-ideality effects according to calculations derived from [60]. Errors were calculated using a Monte Carlo analysis with 95% confidence limit for the determined dissociation constants.

**Supplemental references**

**57.** Droescher M, Begitt A, Marg A, Zacharias M, Vinkemeier U. Cytokine-induced paracrystals prolong the activity of signal transducers and activators of transcription (STAT) and provide a model for the regulation of protein solubility by small ubiquitin-like modifier (SUMO). J. Biol. Chem*.* 2011; 286: 18731-46.

**58.** Meyer T, Begitt A, Lödige I, van Rossum M, Vinkemeier U. Constitutive and IFN-γ-induced nuclear import of STAT1 proceed through independent pathways. EMBO J. 2002; 21: 344-54.

**59.** Zacharias DA, Violin JD, Newton AC, Tsien RY. Partitioning of lipid-modified monomeric GFPs into membrane microdomains of live cells. Science 2002; 296: 913-6.

**60.** Schuck P. On the analysis of protein self-association by sedimentation velocity analytical ultracentrifugation. Anal. Biochem. 2003;320: 104-24.

**61.** Wills PR, Scott DJ, Winzor DJ. Allowance for the effects of thermodynamic nonideality in sedimentation equilibrium distributions reflecting protein dimerization. Anal. Biochem. 2012; 422: 28-32.
